# Valorizing the biosorptive potential and bioactivity of *Anabasis articulata* Sahara plants

**DOI:** 10.1038/s41598-026-66204-x

**Published:** 2026-08-20

**Authors:** Magda A. Akl, Asmaa A. Serage, Aya G. Mostafa, Ahmed I. Abd-Elhamid, Yasser Al-Amier

**Affiliations:** 1 Department of Chemistry, Faculty of Science, Mansoura University, Mansoura, 35516 Egypt; 2 Botany Department, Faculty of Science, Mansoura University, Mansoura, 35516 Egypt; 3 Composites and Nanostructured Materials Research Department, Advanced Technology and New Materials Research Institute (ATNMRI), City of Scientific Research and Technological Applications (SRTA-City), Alexandria, New Borg Al-Arab, 21934 Egypt

**Keywords:** Egypt, Sahara plants, *Anabasis articulate*, Biosorption, Lead(ii), Error functions, Wastewater, Biological activity, Chemistry, Environmental sciences

## Abstract

Finding new affordable biosorbents for the remediation of toxic inorganic pollutants is a great challenge. This challenge arises from the need for biosorbents with functional groups that can efficiently chelate heavy metals and facilitate their removal from water. The present study examines the application of the local Egyptian Sahara medicinal plant *Anabasis articulata*, in both raw (ANA) and NaOH-modified (ANAS) forms, as biosorbents for removing Pb(II) ions from aqueous solutions. The characterization of both biosorbents was conducted using multiple analytical techniques, such as Fourier-transform infrared spectroscopy (FT-IR), Brunauer–Emmett–Teller (BET) surface area analysis, Boehm titration, scanning electron microscopy (SEM), and thermogravimetric analysis (TGA). The effects of experimental variables on Pb(II) biosorption, including initial Pb(II) concentration, pH, biosorbent dosage, contact duration, and the presence of foreign ions, were systematically investigated. To ensure the reliability of the kinetic and isotherm adsorption models, error functions, including χ^2^, SSE, and MSE, were used as validation tools. Analysis of adsorption equilibrium data demonstrated that the Langmuir model most accurately described the process, yielding maximum capacities of 76.38 ± 0.26 mg/g and 46.74 ± 0.21 mg/g for ANA and ANAS, respectively. A measurable enhancement in adsorption was observed following mercerization, which altered the dominant Pb(II) binding mechanism. Thermodynamic analysis revealed that Pb(II) biosorption on ANA and ANAS was exothermic and endothermic, respectively. The ANA extract exhibited significant antioxidant activity as determined by the DPPH radical scavenging assay. Furthermore, the methanolic extract of ANA showed potential antimicrobial activity against both Gram-negative and Gram-positive bacteria. The mechanism of Pb(II) biosorption onto ANA and ANAS biosorbents is elucidated. The ANAS biosorbent, produced via a simple, environmentally friendly process, offers a sustainable solution for wastewater treatment in arid regions.

**Supplementary Information:**

The online version contains supplementary material available at 10.1038/s41598-026-66204-x.

## Introduction

Currently, water pollution caused by heavy metals resulting from increased industrial activity poses a significant environmental challenge. Their ions are very stable and considered persistent environmental pollutants. Moreover, these ions may adversely affect aquatic organisms and represent a significant public health concern^1^. Many polluted water sources host marine organisms that humans eat and can bioaccumulate through the food chain. Arsenic, zinc, cadmium, lead, chromium, and nickel, in addition to copper, are the major contaminants found in water^2^. Since freshwater resources are becoming increasingly scarce, reusing wastewater through various treatment methods helps maintain ecosystem health, prevent water shortages, and protect potable water resources^3^.

Lead, a heavy metal, has a fatal influence on animals and plants as well as causes several diseases in humans^4^. Moreover, certain enzymes and proteins have thio, oxo, and phosphate groups, to which Pb(II) has a high affinity^4^. The Pb(II) binding to those enzymes can alter cellular membrane permeability and affect hemoglobin synthesis. Also, it can bioaccumulate in bones over a lifetime of more than 20 years^4^. The World Health Organization (WHO) has drawn a strict line, allowing no more than 0.01 mg/L of Pb(II) ions in drinking water to protect public health^5^. The FAO/WHO (2001) established a maximum allowable limit of 0.3 mg/kg for Pb(II) in fruits and vegetables on a dry-weight basis^6^. Therefore, before discharging polluted water into the environment and water bodies, it is essential to release Pb(II) from it.

Nowadays, a wide range of wastewater treatment technologies is needed, including chemical precipitation^7^, biosorption^8^, membrane filtration^9^, coagulation-flocculation^10^, adsorption^11^, flotation^12^, and electrochemical treatment^13^.

Biosorption is a biotechnology used to remove heavy metals and their associated organic compounds from waste streams. In addition, it unlocks the potential of diverse agricultural and industrial biomasses, transforming them into powerful biosorbents for cleaner water^14^. It has various advantages, including its effectiveness in water treatment by reducing concentrations and its use of inexpensive materials. Compared to conventional techniques used for heavy metals remediation, biosorption is a quick, reversible, independent of the physiological limitations of living cells, an affordable, and eco-friendly technology^15^. However, there are several drawbacks, including the need to desorb the metal from the material for reutilization, regardless of its value, and the inability to control biosorbent properties through biology^16^. Plants, such as agricultural waste materials, have been used as biosorbents; these processes serve as a means of recycling waste. Consequently, employing these individuals does not incur significant expenditures. The presence of carboxylic and phenolic functional groups in the cellulosic matrix or in components associated with cellulose, lignin, and hemicelluloses is considered the primary reason for the potential of plant-derived biosorbents. To remove water pollutants, such as heavy metal ions, many biosorbents have been utilized. These include macroalgae, microalgae, aquatic plant wastes, and terrestrial plant biomasses. Additionally, agricultural wastes such as fruit peels, cereal straw, and shells, as well as animal-derived materials like hair and crustacean shells, have been investigated for this application^17–19^. The use of biosorbents provides economic benefits as they are low-cost, readily available in large amounts, and easily renewable^20^.

*Anabasis articulata* (ANA), which is widely used in traditional medicine, belongs to the Chenopodiaceae family^21^. The *Anabasis* genus contains saponins and alkaloids, such as *anabasine*, *anabasamine*, *lupinine*, *jaxartinine*, and *triterpenic* sapogenins.

The prospect of the raw (ANA) and NaOH-modified (ANAS) forms of *A. articulata* as cost-effective, environmentally sustainable biosorbents for removing Pb(II) from aqueous solutions under optimal conditions is evaluated. To date, the mercerization of *A. articulata* using NaOH has not been documented in the literature. Additionally, this work represents the first application of ANA and ANAS biosorbents for Pb(II) removal from actual contaminated water samples.

Herein, a biosorbent with inherent bioactivity was used to remove Pb(II) from various wastewater sources. While the main goal of the investigation is biosorption, bioactivity was also demonstrated as an added value that could support broader environmental and antimicrobial applications. Given that the prepared material’s surface functionalities not only enable efficient heavy metal removal but also confer antimicrobial potential, expanding its application scope, this work aims to establish a link between the material’s adsorption performance and its biological activities.

The current study was conducted with the following criteria in mind, based on the previously provided data: (i) *A. articulata* (ANA) desert plants are used to prepare biosorbents in both their raw and modified NaOH forms; (ii) BET, FTIR, surface functional groups applying the Boehm titration approach, SEM, and thermal analysis (TGA and DTG) were utilized to characterize the produced biosorbents; (iii) Examining the many experimental factors influencing Pb(II) biosorption onto biosorbents made from desert plants, (iv) Thermodynamic, kinetic, and isothermal parameters are depicted; v. Investigation of the antioxidant and antibacterial bioassay of *A*. *articulata (*ANA) methanolic extract; (vi) Clarification of the processes by which Pb(II) biosorbs onto the produced biosorbents and the antioxidant mechanism of the methanolic extract of *A.articulata* (ANA) raw materials.

## Experimental

### Materials and solutions

All chemicals and reagents used were obtained from Merck and were of analytical grade. By dissolving Pb(NO_3_)_2_, 331.2 g/mol, in deionized water, a 1000 mg/L stock solution of the Pb(II) was prepared. To restrict hydrolysis, the Pb(II) stock solution is acidified using a small amount of concentrated HNO_3_. After preparing various working dilutions from 1000 mg/L bulk stock solutions, a batch adsorption technique is performed. The pH of the prepared Pb(II) solutions was adjusted with 0.1 N HCl or 0.1 N NaOH.

### Instrumentation

The pH of the prepared solutions was determined using a pH meter (Hi 931401, HANNA, Portugal). Adsorption experiments were conducted with a water bath shaker (NE5, Nickel-Electro Ltd., UK). An analytical balance is used in batch experiments for weighing different materials under study. The concentration of residual Pb(II) was determined using a Varian AA 240Z Graphite Furnace Atomic Absorption Spectrometer (Palo Alto, CA, USA) (GFAAS). Detailed instrumental settings are listed in Table S1.

### Preparations of biosorbents

#### Preparation of raw *Anabasis articulata* (ANA) biosorbent

The aerial parts of the ANA plant (Fig. S1) were collected from naturally occurring populations in Wadi Ash-Shaykh, located in the North Eastern Desert of Egypt (28° 40′ 4.67″ N 31° 3′ 58.47″ E). The identification of *Anabasis articulata* (Forssk.) Moq. was taxonomically identified by Dr. Yasser El-Amier, Assistant Professor of Plant Ecology, Faculty of Science, Mansoura University, Egypt, according to the flora books of Egypt^22^. A voucher specimen (Voucher No. Mans. 0030101004) has been deposited in the Herbarium of the Department of Botany, Faculty of Science, Mansoura University, Egypt. Plant collection was conducted in accordance with the applicable institutional and national regulations. As the sampling site was a non-protected area and *A. articulata* is not subject to collection restrictions at the study site, no specific collection permit or license was required. The field study complied with the relevant institutional, national, and international guidelines for research involving plant materials. Specimens were washed repeatedly with deionized water to remove impurities, air-dried in complete darkness for 1 week, and then ground into a fine powder.

#### Preparation of NaOH-modified *A. articulata* (ANAS) biosorbent

**Fig. 1:**
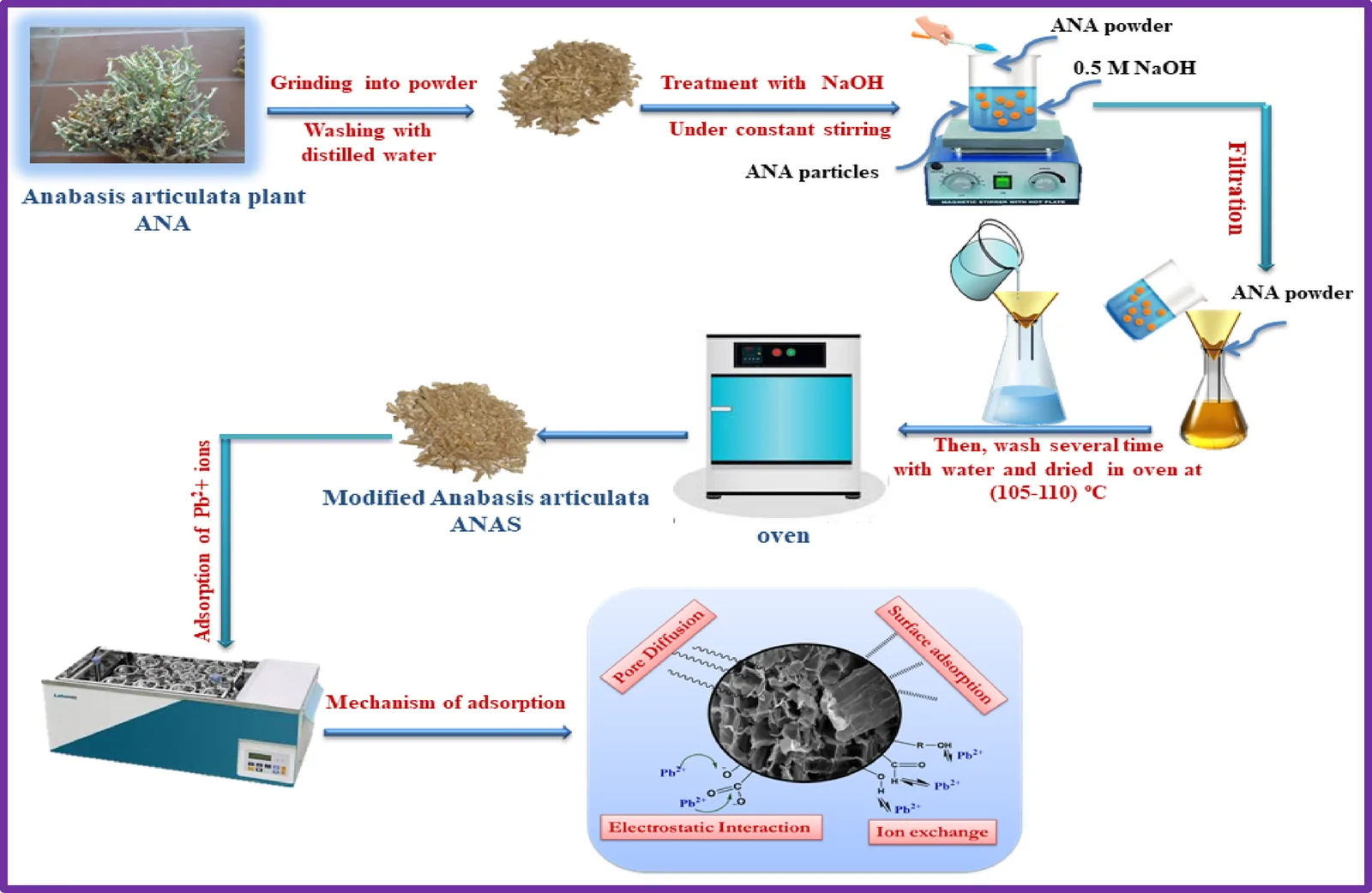
The synthesis steps of ANAS biosorbent.

The ANAS biosorbent was prepared from the obtained raw (ANA) sample, which was modified by soaking (30 g) in roughly 1L of 0.5 M NaOH solution, and continuously stirred for 24 h. The modified biomass (ANAS) was repeatedly decanted and filtered, then rinsed with double-distilled water until the solution pH reached 7.0. Then, it was dried in an oven set to 105–110 °C until it was fully dry. Here, the sample is ready for use. The synthesis steps for ANAS are presented in Fig. 1.

### Characterization

Characterization of the prepared biosorbents is conducted based on their chemical and physical properties.

#### Nitrogen adsorption–desorption analysis

To precisely measure the specific surface areas (m^2^/g) of ANA and ANAS, we employed a Surface Area and Pore Size Analyzer (Quantachrome, NOVA 2000 Series) using the Brunauer–Emmett–Teller (BET) method. This technique is based on N_2_ adsorption–desorption measurements at its boiling point of 77 K.

#### Boehm titration

Using the Boehm titration approach, the ANA surface chemistry, in addition to the content of acid–base functional groups, was revealed^19^. This approach involves neutralizing surface functionalities based on their acid strength. A functional group with a specific pKa can be neutralized only by a base possessing a higher pKa value. A 0.1 g portion of the ANA sample was introduced into four separate bottles, each containing 0.1 M solutions of NaOH, Na_2_CO_3_, NaHCO_3_, or HCl. The bottles were then shaken for 48 h. Approximately 5 mL of each supernatant sample was titrated with HCl and NaOH, enabling precise measurement of their chemical properties. Acidic sites were estimated by noting that NaHCO_3_ neutralizes only carboxylic groups, Na_2_CO_3_ neutralizes carboxylic and lactonic groups, and NaOH neutralizes carboxylic, lactonic, and phenolic groups. By measuring the HCl consumed by the ANA sample, we determined the number and concentration of its basic sites.

#### Determination of surface pH of biosorbents

Initially, approximately 0.1 to 0.5 g of the ANA/ANAS biosorbent was shaken in 25 mL of pre-boiled deionized water for approximately 2 days, after which the supernatant pH was measured. The pH_PZC_ (point of zero charge) of ANA and ANAS was estimated using the solid-addition method^19^. pH_PZC_ was determined by agitating 0.1 to 0.15 g of ANA with 0.01 M NaCl solutions, which were adjusted to pH values in the range of 2–12 using 0.05 M HCl/NaOH. The final pH was measured after 48 h. The initial pH (pH_i_) was plotted against the change in pH (∆pH, defined as pH_i_ minus pH_f_). At the point of zero charge (pH_PZC_), the solution pH remains constant during the equilibration period^23^.

#### Ash and moisture content

The amount of minerals present in the samples as impurities is measured by ash content. It was determined that various doses of 0.3–0.5 g of dried ANA were put in a muffle furnace for about 6 h at a temperature of (700–800)°C, and then the ANA’s final weight was recorded. The ANA ash content (%), Eq. 1, was assessed utilizing the ANA sample weight before heating and its weight after heating^19^:

1 $${\mathrm{Ash}}\,{\text{content \% }} = \frac{{{\mathrm{Final}}\,{\text{weight }}\left( {\mathrm{g}} \right)}}{{{\mathrm{Initial}}\,{\text{weight }}\left( {\mathrm{g}} \right)}} \times 100$$

Moisture content (%), Eq. 2, is the amount of adsorbed water in the ANA sample when stored under humid conditions. Certain biosorbents may adsorb about 25:30% moisture and also seem dry. Consequently, to provide the necessary dry weight, an additional weight of moist samples is required. The sample (0.3–0.5 g) was heated at 110 °C for 24 h, then cooled to room temperature in a desiccator and reweighted. The moisture content is calculated using the following equation^19^.

2 $${\mathrm{Moisture}}\,{\text{content \% }} = \frac{{{\mathrm{Loss}}\,{\mathrm{in}}\,{\text{weight }}\left( {\mathrm{g}} \right)}}{{{\mathrm{Initial}}\,{\mathrm{weight}}}} \times 100{ }$$

#### Scanning electron microscopy (SEM)

The morphology of ANA and ANAS was examined with a JSE-T20 (JEOL, Japan) scanning electron microscope at an acceleration voltage of 40,000 V.

#### FT-IR analysis

KBr-pressed discs were used to obtain FTIR spectra of biosorbents prepared using a Shimadzu 5800 Fourier FTIR spectrometer. Data obtained from the Jasco instrument (Model 6100, Japan) ranged from 4000 to 400 cm^-1^.

#### Thermal studies (thermo gravimetric analysis)

The thermal stability of ANA and ANAS was analyzed utilizing a Thermo-Gravimetric Analyzer (Shimadzu, Japan) at a heating rate of 10 °C/min from 25 to 800 °C under a nitrogen gas flow.

**Fig. 2:**
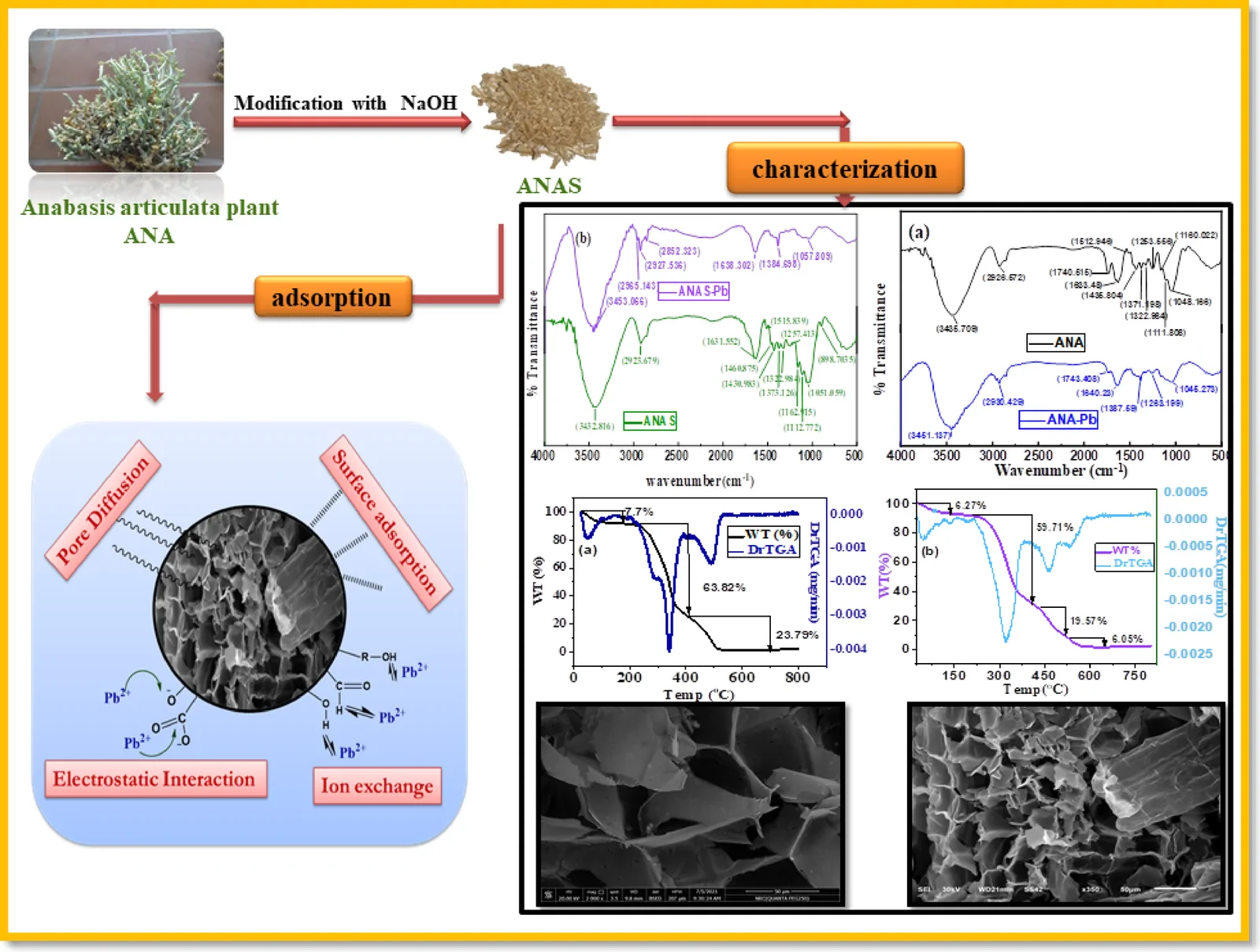
The synthesis steps, characterization of ANA and ANAS biosorbents, and the mechanism of biosorption of Pb(II) onto ANA and ANAS biosorbents.

The synthesis steps, characterization of ANA and ANAS biosorbents, and the Pb(II) biosorption mechanism onto ANA and ANAS biosorbents are schematically represented in Fig. 2.

### Methodology

#### Pb(II) adsorption batch tests

Experiments were conducted in 125 mL bottles with a fixed biosorbent dosage of 1 g/L, while the Pb(II) concentration in 25 mL of solution was varied from 10 to 300 mg/L. The mixtures were agitated at 150 rpm at room temperature. The biosorbents ANA/ANAS were subsequently removed by centrifugation. GFAAS analyzed the remaining Pb(II) concentrations in the resulting supernatant solutions. Various effects, including pH levels between 1 and 6 for ANA and 1.5 to 6 for ANAS, doses from 0.01 to 0.09 g, temperature ranging from 25 °C to 60 °C, and contact time from 1 to 300 min, were studied for the adsorption of Pb(II) onto the surface of ANA and ANAS biosorbents.

##### Effect of pH

The pH of an aqueous solution significantly influences biosorption by affecting the biosorbent surface charge, ionization, and adsorbate speciation. This study examined pH to identify optimal conditions for maximum biosorption capacity.

To determine the optimum pH for biosorption, 0.025 g of ANA/ANAS was shaken in 25 mL of a 50 mg/L Pb(II) solution at pH values from 1 to 6 for ANA and 1.5–6 for ANAS, adjusted with 0.1 M NaOH or 0.1 M HCl^24^. Samples were shaken at 25 °C for 5 h. The pH value is plotted against adsorption capacity (q_e_).

##### Influence of initial concentration of Pb(II)

The equilibrium isotherm of Pb(II) was evaluated by varying its initial concentration from 10 to 300 mg/L, agitating 0.025 g of ANA and ANAS samples in 25 mL of solution at room temperature for 6 h^25^. Maximum biosorption capacity and adsorbate removal were calculated using Eqs. (3) and (4).

3 $${\mathrm{q}}_{{\mathrm{e}}} = \frac{{\left( {{\mathrm{C}}_{{\mathrm{o}}} - {\mathrm{C}}_{{\mathrm{e}}} } \right){\mathrm{V}}}}{{\mathrm{W}}}$$

4 $${\text{R\% }} = \frac{{\left( {{\mathrm{c}}_{{\mathrm{o}}} - {\mathrm{c}}_{{\mathrm{e}}} } \right)}}{{{\mathrm{c}}_{{\mathrm{o}}} }}x100$$

where C_o_ and C_e_ are the initial and equilibrium Pb(II), V is the volume of Pb(II) solution (L), and W is the weight of the biosorbent (g). Moreover, the percentage removal of Pb(II) from aqueous solution is expressed as R (%).

##### Effect of contact time

Contact time was studied over 1–300 min to evaluate adsorption kinetics^26^. For each trial, 0.025 g of Sahara plants was added to bottles containing 25 mL of a 50 mg/L solution at a constant pH of 4.5–5. Kinetic models, including pseudo-1^st^-order, pseudo-2^nd^-order, intraparticle diffusion, and Boyd models, were assessed for Pb(II) adsorption using the ANAS biosorbent based on these experimental results.

##### Error validity of adsorption isotherm and kinetic models

Identifying the most appropriate model for the experimental data obtained from the adsorption methodology is a very important step. R^2^ alone is insufficient to determine which model best describes adsorption in the Sahara plant under study. Here, we applied several error functions to determine which model best explains adsorption processes in isotherm and kinetic studies. These functions are the chi-square statistic (${x}^{2}$, Eq. 5), mean square error (MSE, Eq. 6), hybrid fractional error function (HYBRID, Eq. 7), Sum of squares of the errors (SSE, Eq. 8), and normalized standard deviation (Eq. 9).

The chi-square statistic^27^:

5 $${\upchi }^{2} = \mathop \sum \limits_{{{\mathrm{i}} = 1}}^{{\mathrm{n}}} \frac{{({\mathrm{q}}_{{{\mathrm{e}},{\mathrm{exp}}}} - {\mathrm{q}}_{{{\mathrm{e}},{\mathrm{calc}}}} )^{2} }}{{{\mathrm{q}}_{{{\mathrm{e}},{\mathrm{cal}}}} }}$$

Mean square error^27^:

6 $${\mathrm{MSE}} = \frac{1}{{\mathrm{n}}}\mathop \sum \limits_{{{\mathrm{i}} = 1}}^{{\mathrm{n}}} (({\mathrm{q}}_{{{\mathrm{e}},{\mathrm{exp}}}} - {\mathrm{q}}_{{{\mathrm{e}},{\mathrm{calc}}}} )^{2}$$

Hybrid fractional error function (HYBRID)^26^:

7 $${\mathrm{HYBRID}} = \frac{100}{{\text{N - P}}}\sum\nolimits_{{\text{i = 1}}}^{{\mathrm{n}}} {} \left[ {\frac{{{\mathrm{(q}}_{{\mathrm{e,exp}}} - {\mathrm{q}}_{{\mathrm{e,calc}}} {)}^{{2}} }}{{{\mathrm{q}}_{{\mathrm{e,exp}}} }}} \right]$$

Sum of squares of the errors^27^:

8 $${\mathrm{SSE}} = \mathop \sum \limits_{{{\mathrm{i}} = 1}}^{{\mathrm{n}}} ({\mathrm{q}}_{{{\mathrm{e}},{\mathrm{exp}}}} - {\mathrm{q}}_{{{\mathrm{e}},{\mathrm{calc}}}} )^{2}$$

Normalized standard deviation^28^:

9 $$\Delta {\text{q\% }} = 100\sqrt {\frac{{\sum \left( {\frac{{{\mathrm{q}}_{{{\mathrm{e}},{\mathrm{exp}}}} - {\mathrm{q}}_{{{\mathrm{e}},{\mathrm{calc}}}} }}{{{\mathrm{q}}_{{{\mathrm{e}},{\mathrm{exp}}}} }}^{2} } \right)}}{{{\mathrm{n}} - 1}}}$$

In this context, q_e_,_exp_ refers to the experimentally determined amount of Pb(II) adsorbed (mg/g) per gram of ANA/ANAS biosorbent, whereas q_e_,_cal_ denotes the amount calculated by the model (mg/g). The variables n and p denote the number of experimental observations and the number of model parameters, respectively.

##### Effect of temperature

To study the effect of temperature on adsorption, 25 mL of adsorbate solution with 0.025 g of biosorbent at 100 mg/L was shaken at temperatures between 25 and 60 °C^29^. Thermodynamic parameters (ΔG°, ΔH°, and ΔS°) were then determined.

##### Effect of biosorbent dose

To determine the optimal dosage of the adsorbent for Pb(II) removal from the prepared samples, 25 mL of a 50 mg/L Pb(II) solution was shaken with different doses of biosorbent ranging from 0.01 g to 0.09 g for 24 h at room temperature.

##### Effect of foreign ions

The effect of foreign ions was studied by shaking 0.025 g in 25 mL of 20 mg/L of Pb(II) containing 100 mg/L of different cations and anions that may be present in drinking water. These prepared mixtures were shaken for 24 h, and the GFAAS analyzed the suspensions.

##### Effect of ionic strength

In addition, the effect of ionic strength by adding NaCl was studied on the removal of Pb(II), by shaking 0.025 g of ANA/ANAS biosorbent in a 25 mL solution containing 20 mg/L of Pb(II) at NaCl concentration ranging between 1 × 10^–3^ M and 0.15 M at room temperature for 24 h, and then the suspensions were analyzed^19^.

### Sample analysis

Using varying adsorbate concentrations, the biosorption of Pb(II) ions from various environmental samples, including deionized water, ground water, tap water, and various food samples, was investigated. The investigated water samples’ organic matter was digested as follows: a mixture of 0.5 g K_2_S_2_O_8_ and 5 mL of H_2_SO_4_ (95.0–98.0% (w/w)) was added to each water sample, followed by heating at 90 oC for 2 h. For food samples, they were first digested with concentrated nitric acid at high pressure and temperature. 0.025 g of biosorbent was shaken with different Pb(II) ion concentrations in various environmental samples for 24 h, then filtered. After that, another 0.025 g of biosorbent was added to the supernatant to ensure the complete separation of analytes. Atomic absorption spectroscopy was then used to evaluate the filtrate and find the equilibrium concentration of Pb(II) ions.

### Desorption study

A desorption investigation was conducted to evaluate the prepared biosorbents’ ability to desorb Pb (II) from biosorbed Pb(II) and to reutilize ANA and ANAS biosorbents further. To remove any unadsorbed materials, the adsorbents were gently washed with water after adsorption. At 105–110 °C, the samples were dried in an oven. Then, the plant’s known weight (0.025 g) was shaken with varying pH levels. Solutions that were adjusted from 1.5 to 12 using (HCl and NaOH) solutions^19^. The adjusted pH solutions after shaking were filtered, and the Pb(II) desorption (%) from ANA and ANAS was estimated as presented in Eq. (10):

10 $${\mathrm{De}} - {\mathrm{sorption}}\% = \frac{{{\text{Amount }}\,{\mathrm{desorped}}\,{\mathrm{to}}\,{\mathrm{the}}\,{\mathrm{solution}}\,\left( {{\mathrm{mg}}/{\mathrm{l}}} \right){ }}}{{{\mathrm{Amount}}\,{\mathrm{adsorped}}\,{\mathrm{to}}\,{\mathrm{the}}\,{\mathrm{solution}}\,\left( {{\mathrm{mg}}/{\mathrm{l}}} \right)}} \times 100$$

### Bioactivity studies

#### Antioxidant activities

As reported by Kosem et al.^30^, ANA antioxidant activity was assessed using an ANA methanolic extract, with some modifications. A volume of 0.2 L of methanol (50%) was used to extract about 20 g of powdered samples, which were left at room temperature for a week. The ANA methanolic extract was then collected and vacuum-filtered in a Buchner funnel using Whatman No. 1 filtering paper. Various concentrations were then used.

*DPPH free radical scavenging activity. *Employing an approach based on Lim and Quah^31^, the scavenging activity of the DPPH (2,2-diphenyl-1-picrylhydrazyl) stable radical was utilized to evaluate a compound’s capacity to donate a hydrogen atom. To 1 mL of plant extract at concentrations in the range of (5–50 mg/mL), 2 mL of 0.15 mM DPPH were added. 2 mL of 0.15 mM DPPH was added to 1 mL of solvent (50% methanol) to provide a control. After mixing the tube contents and allowing them to settle for 30 min, the absorbance at λ_max_ 517 nm was measured.

The antioxidant activity was expressed in Eq. (11):

11 $${{\% }}\,{\mathrm{radical}}\,{\mathrm{scavenging}}\,{\mathrm{activity}} = \left[ {1 - { }\left( {{\mathrm{A}}_{{{\mathrm{sample}}}} /{\mathrm{A}}_{{{\mathrm{control}}}} } \right)} \right] \times 100$$

where absorbance at 517 nm is presented by A.

Additionally, the IC_50_ was determined as the ANA plant amount (mg) in 1 mL of solution required to reduce the DPPH initial concentration to half its initial value. For comparison, the antioxidant activity of ascorbic acid was also measured in the concentration range of (1–20 mg/mL).

#### Antimicrobial bioassay

Before the grinding step, the ANA plant sample was thoroughly cleaned, repeatedly rinsed with distilled water, and then dried in a forced-air oven at 55–60 °C for up to 24 h. 100 g of dried ANA plant powder was extracted separately with water, petroleum ether, and 95% methanol, and soaked overnight with frequent shaking. Filter paper with an 11 µm medium flow was used to filter the ANA extract, which was then dried by evaporation. Dimethyl sulfoxide (DMSO) was used to dissolve the ANA dry residue, which was then stored at − 20 °C for further use^32^.

- Tested bacteria

The microbial species were purchased from the Cairo Microbiological Resources Center (Cairo MIRCEN) at the Faculty of Agriculture, Ain Shams University, Egypt. The Gram-negative bacteria used were as follows: Escherichia coli (ATCC 10536), *Pseudomonas aeruginosa* (ATCC 9027), Salmonella Typhimurium (ATCC 25566), and *Klebsiella pneumoniae* (ATCC 10028). The Gram-positive bacteria used were as follows: Bacillus subtilis (DMS1088), Staphylococcus aureus (ATCC 6538), and *Staphylococcus pneumoniae* (VGM84130). Cephradine, tetracycline, and ampicillin were used as standard antibiotics.

b) Assay for antibacterial activity

The antibacterial activity of the investigated materials was assessed using a modified Kirby-Bauer disc diffusion method^33^. 5 mm-diameter sterilized filter paper discs were placed on a nutritional agar medium that had been inoculated with the tested pathogenic microorganisms for the antibacterial test after being submerged in the prepared plant extracts overnight^34^. A filter disk was used as a negative control, impregnated with 10 µl of solvent (DMSO). After 24 h of incubation at 37 °C, the inhibition zone diameter (mm) in the Petri dishes was measured.

## Results and discussion

### Alkali modification and physicochemical properties of biosorbents

#### Alkali modification of ANA biosorbent

Although alkali treatment is commonly reported to increase surface area in lignocellulosic biosorbents, the present study showed a slight decrease in the BET surface area after NaOH modification^35^. This reduction may be attributed to partial collapse of the pore structure, pore blockage by residual inorganic species (e.g., Na-containing groups), or structural rearrangement of the cellulose/hemicellulose matrix^36^. The most common method for activating biosorbents is to use heat, vacuum, or superheated steam to desorb the adsorbed gases. Other commonly used methods include roughening the biosorbent’s surface or breaking it into smaller pieces. Additionally, often-utilized chemicals such as NaOH and KOH can be used to activate the biosorbent’s surface. It has been found that the NaOH activation procedure enhances the properties of the biosorbents by increasing total pore volumes and pore diameters. Here, the alkali modification of ANA was carried out at 0.5 M NaOH for 24 h. This may be returned to the elimination, leading to an increase in active functional groups such as C–OH and C–O. In addition, the alkaline treatment increases swelling capacity. Because of its capacity to break down the lignocellulosic materials’ internal structure and enhance their pore structure, NaOH is regarded as the most extensively utilized alkaline agent among a variety of alkaline agents. The OH- ionization to the alkoxide is made possible by treating raw fibers with NaOH^37^. Despite the decrease in surface area, the adsorption behavior was not solely governed by textural properties but also by the availability and nature of active functional groups, particularly oxygen-containing moieties responsible for Pb(II) binding.

In the current study, alkali treatment resulted in noticeable changes in the physicochemical characteristics of ANA, as evidenced by BET, FTIR, Boehm titration, SEM, and TGA analyses. The modification altered the distribution of surface functional groups and pore characteristics of the biosorbent. However, the effect of NaOH treatment on Pb(II) adsorption was not uniformly positive under all investigated conditions, indicating that adsorption performance depends not only on surface area and porosity but also on the nature and abundance of active binding sites involved in Pb(II) uptake.

#### Nitrogen adsorption- desorption analysis

**Fig. 3:**
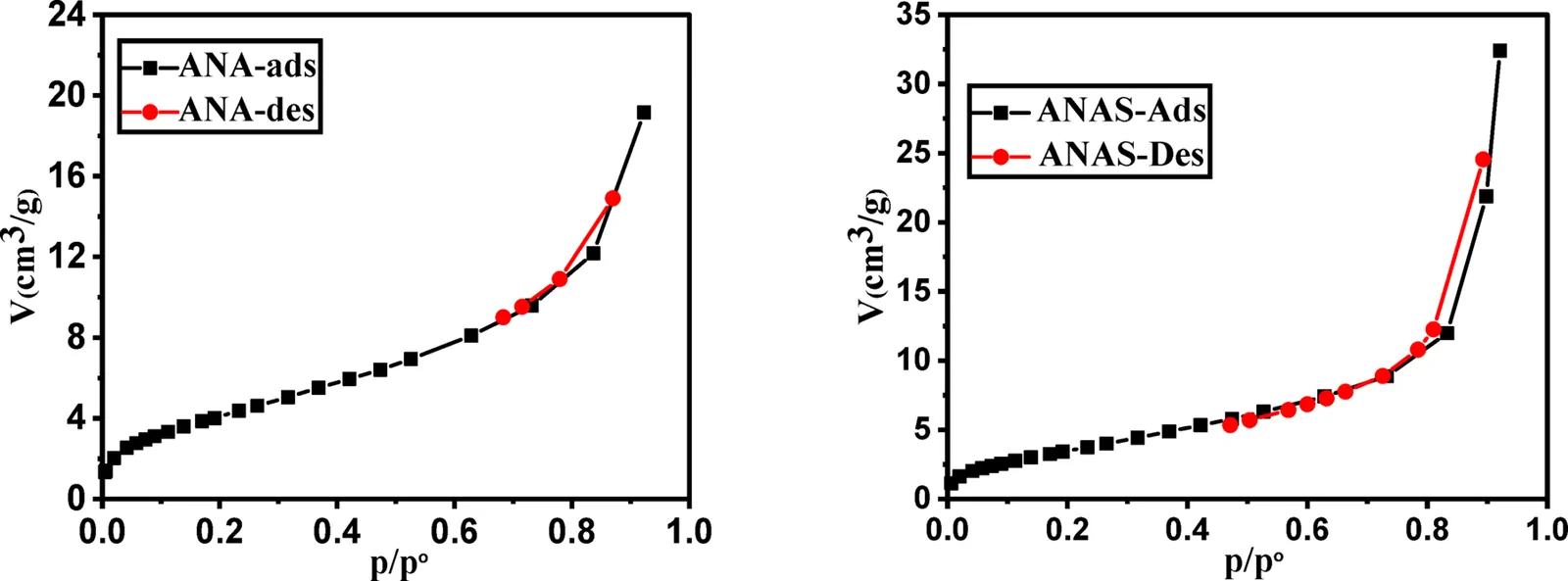
Nitrogen adsorption–desorption isotherms at 77 K for ANA and ANAS biosorbents.

In accordance with Fig. 3**,** the N_2_ adsorption–desorption isotherms for both ANA and ANAS samples indicate that their shapes are Type IV, characteristic of mesoporous materials^38^.

**Fig. 4:**
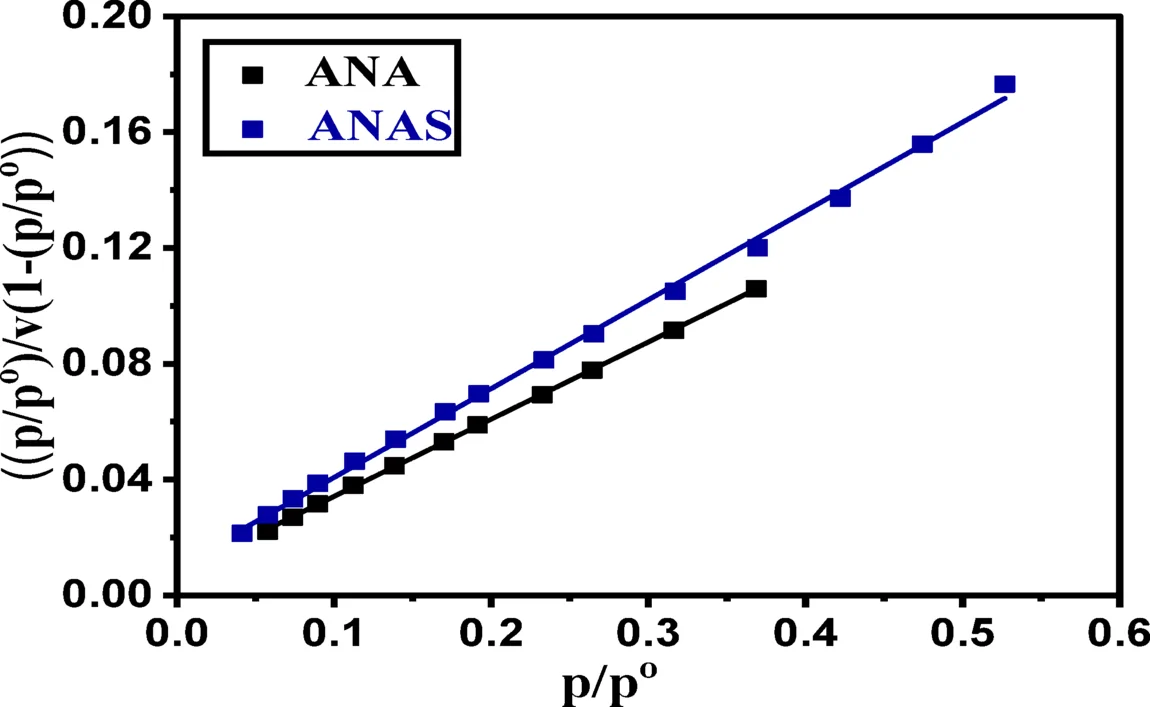
BET linear plots of nitrogen adsorption isotherms at 77 K, ANA and ANAS biosorbents.

Table 1 details all the parameters that were assessed from the N_2_ adsorption–desorption isotherms at 77 K. It showed that the surface area after modification decreases with increasing total pore volume and mean pore diameter. Figure 4 shows the BET linear plots of N_2_ adsorption isotherms at 77 K for ANA and ANAS samples.

**Table 1:** The calculated S_BET_ of prepared ANA and ANAS biosorbents from N_2_ adsorption isotherms.

Sample	ANA	ANAS
S_BET_ (m^2^/g)	16.039	14.39
Total pore volume(cm^3^/g)	0.0296	0.050
Mean pore diameter $(\overline{\mathrm{r})}$	7.391	13.92
Aldehyde content	16%	0.12%

The total mean pore diameters of ANA and ANAS are 73.91 and 139.2 Å, respectively. Compared to the radius of the Pb atom (146 pm), the total mean pore diameters of ANA and ANAS are greater than the radius of the Pb atom. This would allow Pb(II) to diffuse into the pores of the prepared biosorbents readily.

#### Ash and moisture content of the prepared samples

Ash and moisture contents are two critical factors that influence the adsorption characteristics of biosorbents. Ash represents the inorganic, inert, amorphous, and unusable portion found in the activated samples, originating from the raw material. In contrast, ANA/ANAS plant moisture content describes how many H_2_O molecules are normally physically bonded to the ANA biosorbent.

Both ash and moisture can diminish the overall efficacy of the activated sample; thus, a lower ash and moisture content indicates a superior activated sample for adsorption purposes.

Both ash and moisture content can influence the adsorption capacity of activated samples, as well as the point of zero charge, surface pH, and the functional groups present in the ANA plant samples. Table 2 provides details on all the plant surface characteristics.

**Table 2:** Physicochemical characteristics of the ANA & ANAS biosorbents.

Plant samples	% of Moisture content	% of Ash content	Supernatant pH	pH_pzc_	Bohem titration (mmole/g)			
					Carboxylic	Lactonic	Phenolic	Total basic
ANA	7.7	1.66	6.14	6.05	1.05	0.45	1.45	3.05
ANAS	6.27	1.96	6.5	6.35	0.16	1.92	0.17	4.12

The elevated moisture levels ranging from 6.27% to 7.7% in the adsorbent samples, as indicated in Table 2, suggest that the ANA sample has a large surface area and numerous active sites, along with the presence of oxygen-containing functional groups, as confirmed by FTIR and Boehm titration. ANAS has higher ash content than ANA, which could result from sodium introduced into the plant’s structure^19^.

#### Boehm titration

The Boehm titration was used to investigate the acidic and basic properties of *A. articulata*^19^. The concept is based on neutralizing surface functionalities according to their acid strength; a base with a higher pKa can neutralize a functional group with a given pKa.

Data from modified samples in the Boehm titration are documented in Table 2. The amount of basic sites on their surfaces exceeds the number of acidic sites, supporting their basic nature as indicated by pH_pzc_ and pH_sup_ results.

### Characterization

#### SEM

**Fig. 5:**
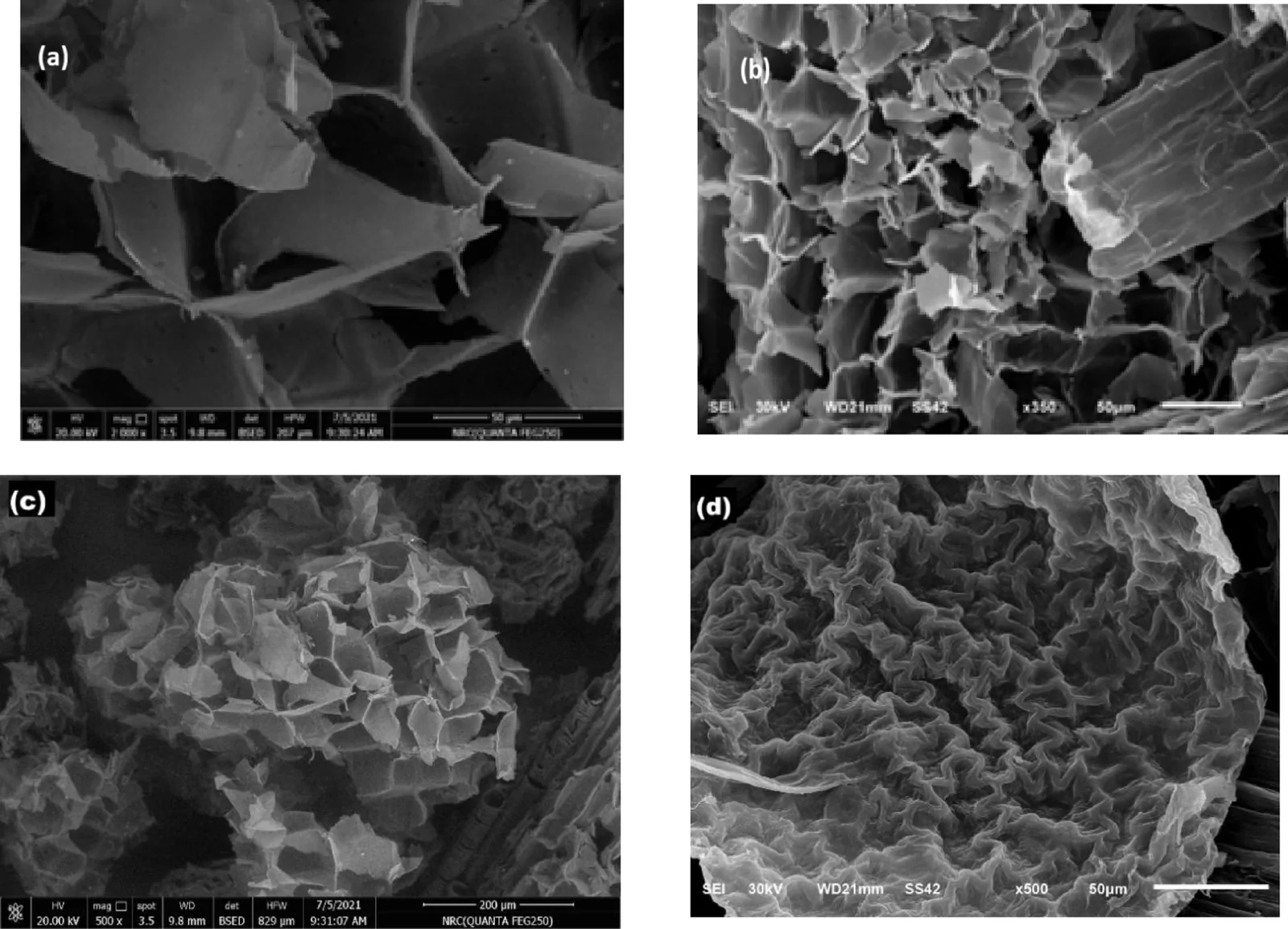
SEM micrograph of plant samples, (**a**) for ANA (× 2000), (**b**) for ANAS (× 350), (**c**) for ANA (× 500), and (**d**) for ANAS (× 500).

SEM was used to examine the morphology of the derived biosorbents. SEM morphology reveals how activation affects the surface morphology of biosorbents. When comparing the surface morphology of the raw *A. articulata* (ANA) plant with that treated with sodium hydroxide (ANAS), a distinct change is evident in the images as presented in Fig. 5a–d^19^.

In Fig. 5, it is noticeable that the biosorbents display a rough texture with a nonuniform surface and a range of randomly distributed pore sizes.

#### FT-IR spectra

The importance of natural compounds in Pb(II) ion removal stems from their containing various functional groups, such as hydroxyl, carboxylic, and carbonyl groups, as well as NH and NH_2_ groups.

**Fig. 6:**
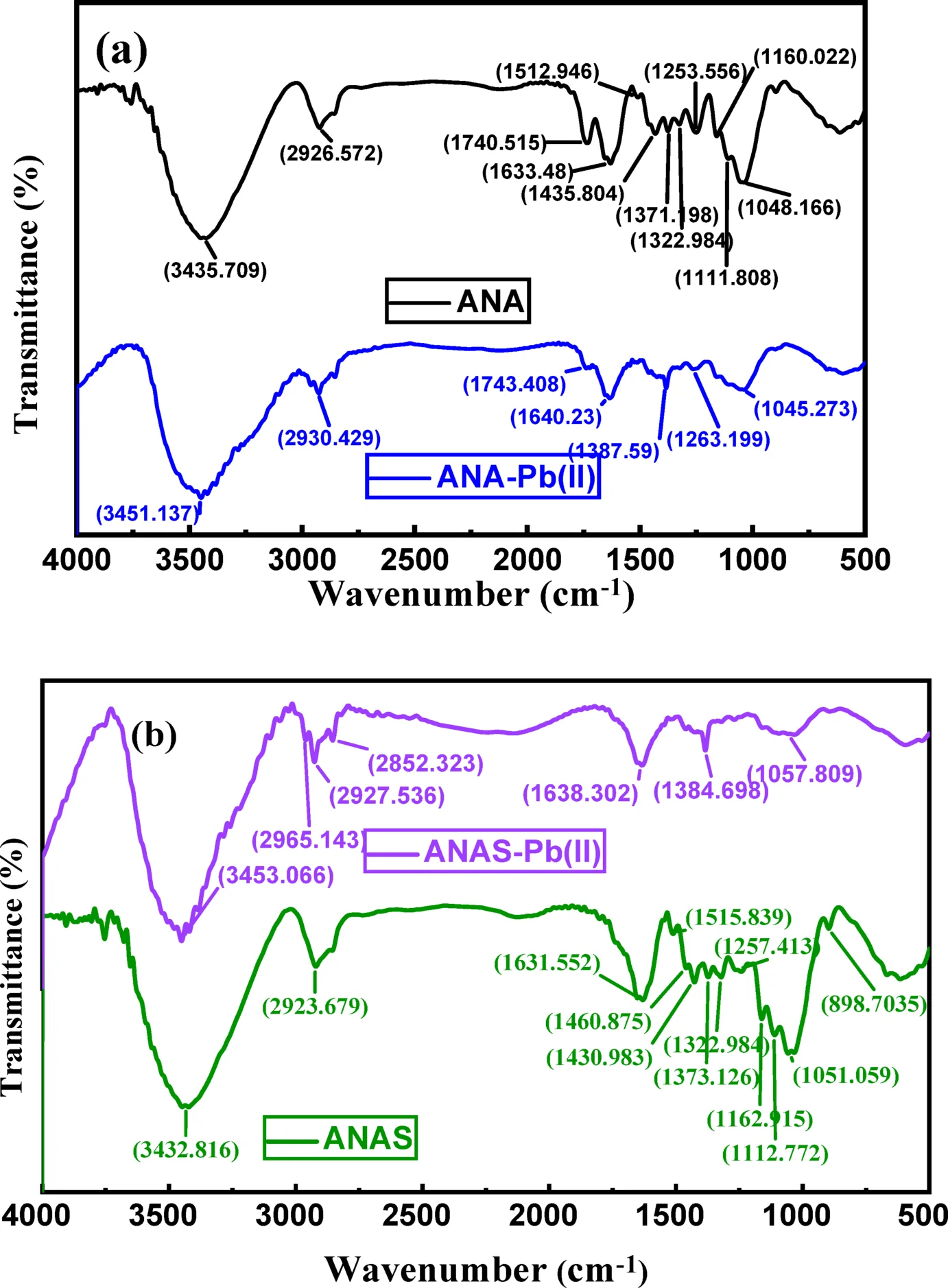
FTIR spectra of (**a**) (ANA and ANA-Pb(II)), (**b**) (ANAS and ANAS-Pb(II)).

The FTIR spectra of both ANA and ANAS Sahara plant after Pb(II) biosorption are shown in Fig. 6. Broad band observed in both samples between (3431 and 3454 cm^-1^) is ascribed to (O–H) hydroxyl groups stretching vibration in alcohol, phenol and adsorbed H_2_O in cellulosic materials in both spectra, and the other weak bands in all samples at (2923–2930 cm^-1^) observed were assigned to aliphatic (C–H) group that may present in such plant^39,40^.

A peak at 1740 cm^-1^ for the ANA sample was attributed to (C=O) stretching vibration of ester and carboxylic acid groups (–COOH). The new peak at 1460 cm^-1^ is attributed to the symmetric CH_3_ group. It is suggested that carboxyl groups (–COOH) are responsible to some extent for the binding of metal ions. This indicates that the biomass’s capability to bind metals can be improved by adding more carboxylate groups^41^.

After modification with NaOH, the peak intensities decreased, and the peak at 1740 cm^-1^, which was present in the ANA sample, disappeared, indicating a decrease in carboxylic content.

A further band can be noticed at the position of 1253.11–1257 cm^-1^, which is attributed to the existence of the lignin (C–O) aryl groups^42^. While at (1371–1373 and 1430–1435 cm^-1^), two peaks were observed, and they represent (C–H) methyl groups^40^ and phenolic (O–H) of ANA plant, respectively^43^. At the (1048–1051 cm^-1^) position, a broad band was observed that may be attributed to the hemicellulose esters’ (C–O) stretching vibration band.

##### FTIR spectra of ANA and ANAS biosorbents after Pb(II) adsorption

As illustrated in Fig. 6a and b, there are various changes in the absorption bands in the FTIR spectra, which confirm the adsorption of Pb(II) by ANA and ANAS samples to a high degree. For both ANA and ANAS biosorbents, the peaks at the positions of (3435 and 3432) cm^-1^ were altered into (3451 and 3453) cm^-1^, respectively. Additionally, the bands at (1633 and 1631) cm^-1^ were changed to other positions at (1640 and 1638) cm^-1^, respectively. Accordingly, adding more carboxylate ligands to the biomass can shift the metal bands at around 1371 cm^-1^ and 1373 cm^-1^ to 1387 cm^-1^ and 1384 cm^-1^, respectively. A band at 1253 cm^-1^ is moved to 1283 cm^-1^ for the ANA sample. Furthermore, the bands at 1048 and 1051 cm^-1^ were repositioned to 1045 and 1057 cm^-1^, respectively. These alterations in both ANA and ANAS peaks after Pb(II) indicate Pb(II) adsorption.

#### TGA

**Fig. 7:**
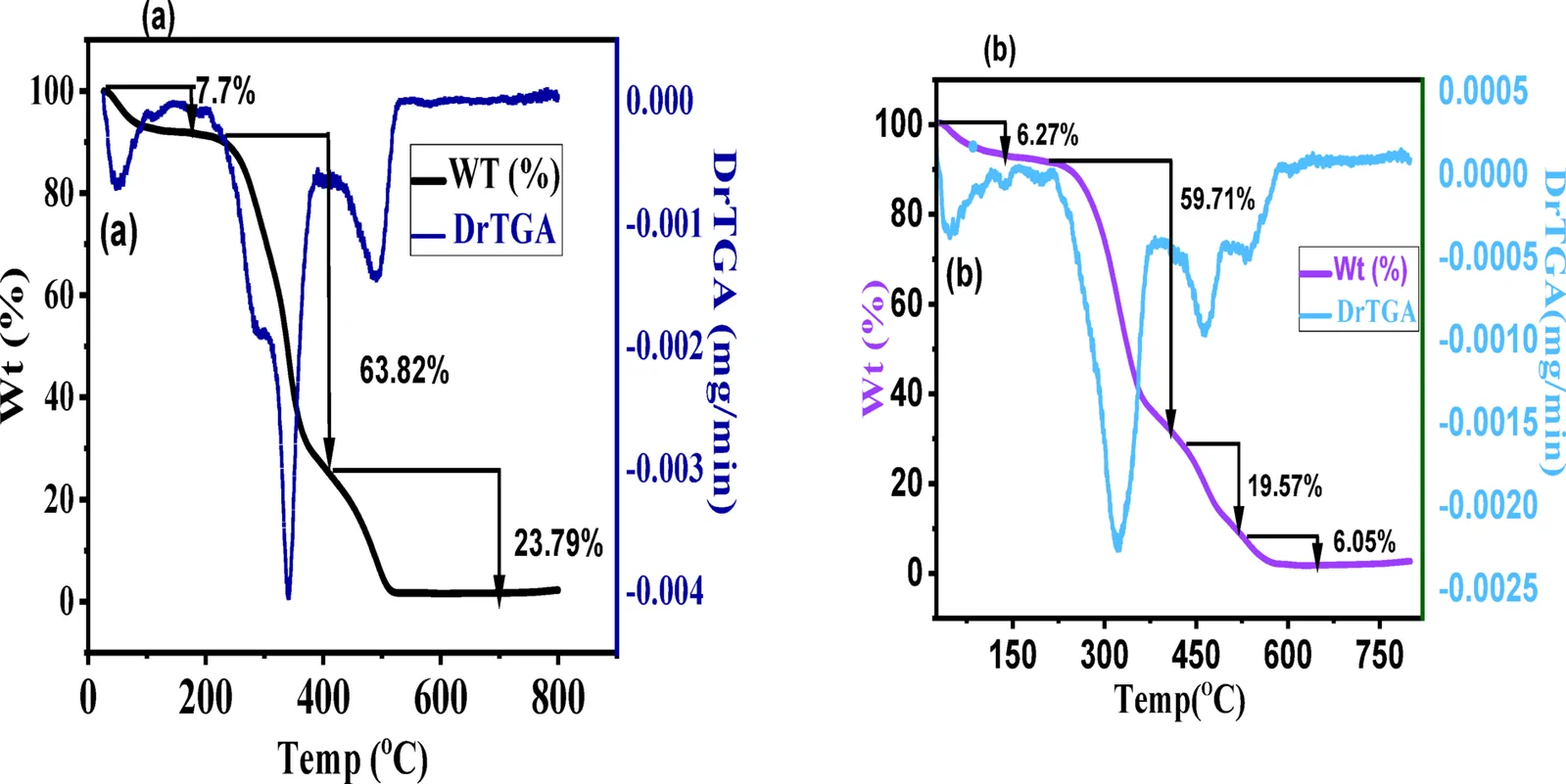
Thermal analysis of (**a**) ANA, (**b**) ANAS biosorbent.

According to the TGA diagram, Fig. 7, the first step reveals weight loss (%) of 7.7 and 6.27% in the (33–177) ^o^C temperature range for ANA and ANAS samples, respectively. This could be explained by the following order of release of moisture and volatile matter found in such plants. The second step shows a gradual weight loss of (63.82 and 59.71%), at temperatures ranging from (217 °C to 410 °C), which may be primarily related to the breakdown of hemicellulose and other low molecular weight materials in the same order. A slight weight loss was observed in the 3^rd^ decomposition step at temperatures above 400 °C, which could be attributed to cellulose deconstruction. Moreover, rising temperatures of more than 700 °C may result in the decomposition of lignin with Weight loss (%) of 23.79% and 19.57% for ANA and ANAS, respectively^19,44^.

### Biosorption studies

#### pH_PZC_ and pH studies

The pH of ANA and ANAS supernatants is crucial for determining the functional groups present on their surfaces. In general, the amphoteric nature and surface charge of ANA and ANAS samples are due to the various functional groups present on their surfaces. The pH_PZC_ of the studied samples is determined when the total ANA/ANAS surface charge equals zero or the pH at which the net number of positive and negative charges on the ANA/ANAS surface becomes zero^27^. It enables the measurement of the surface acidic or basic nature of either the ANA or ANAS sample. The pH_PZC_ and the pH of the solution assess the charge that will be present on the ANA/ANAS surface. At pH above the pH_PZC_, the surface becomes negatively charged due to deprotonation of functional groups, whereas at pH below the pH_PZC_, it becomes positively charged. Several factors, including the charge on the adsorbent surface, influence adsorption characteristics.

The pH_PZC_ of ANA and ANAS samples is determined using the solid-addition methodology^19^. From Table 2, the pH of the supernatant for plant samples was 6.14 and 6.5 for ANA and ANAS, respectively. After activation, ANAS shows a slight increase in pH, which may be due to the return of basic functional groups on the sample surface during the treatment. This result is very close to those reported in the literature. After Alkylation, coffee waste sifted from 4.8 to 6, while cocoa shifted from 6 to 6.8^45^. Akl et al. reported that the *Haloxylon salicornicum* shifted from 6.17 to 6.61 after its treatment with NaOH^19^. Moreover, Farahani et al. reported that sugarcane bagasse pH_PZC_ shifted from 4 to around 6.6 (that is very close to our result^46^.

In solution, the adsorbent surface’s Bronsted acidic groups tend to give up their protons to water molecules, making the surface negatively charged. Lewis bases, on the other hand, become positively charged when protons are removed from solution. The majority of oxygen-containing functionalities are known to act as Bronsted acids, donating protons to the aqueous medium and lowering the pH below 7 in biosorbents^47^. It is known that the fundamental characteristics result from two distinct contributions: surface complexes and proton adsorption on the aromatic basal planes of the plants.

**Fig. 8:**
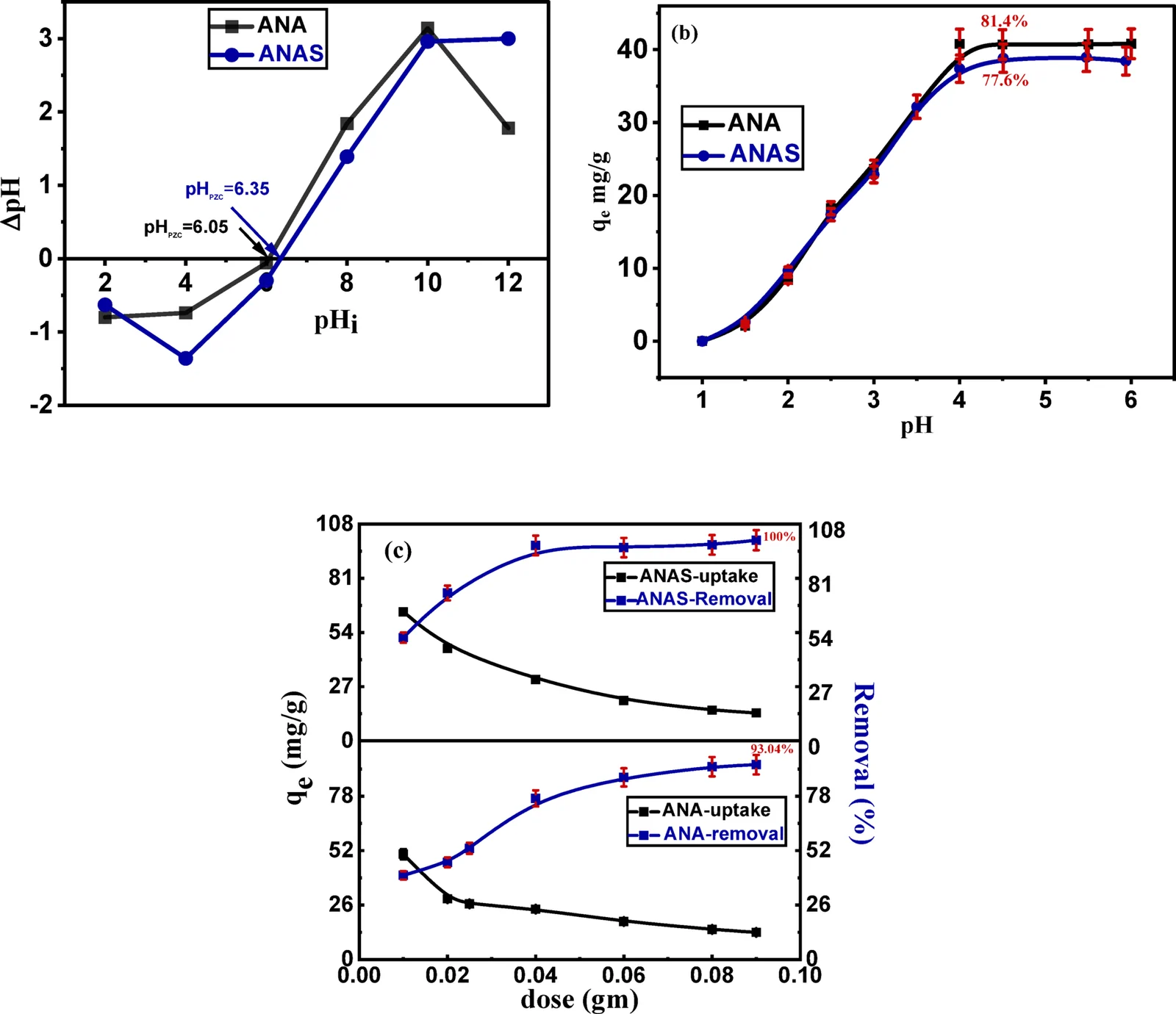
(**a**) Point of zero charge (pH_PZC_); (**b**) Effect of pH on the adsorption capacity of Pb(II) using ANA and ANAS biosorbents (conditions: 0.025 g of ANA/ANAS was taken at 25 mL 50 mg/L of Pb(II) for 5 h); (**c**) Effect of ANA and ANAS dosage for Pb(II) ions removal (conditions: 25 mL 50 mg/L of Pb(II) for 24 h, at pH at room temperature).

So, the surface charge of biosorbents, the pH in aqueous solution, and the amphoteric nature of biosorbents are generally due to the different functionalities present on the biosorbent’s surface. Both Fig. 8a and Table 2 show the ANA and ANAS samples’ point of zero charge (pH_PZC_).

#### Effect of pH

The pH of the aqueous solution is one of the most critical variables that determine adsorption processes, as it controls the biosorbent’s surface charge, ionization state, and speciation. ANA and ANAS Sahara plant samples were used to examine Pb(II) remediation at pH values from 2 to 6.5 to determine how solution pH affects Pb(II) removal. Removal of Pb(II) ions above pH (6.5) is not favored since, in these conditions, lead is detected as the precipitated species Pb(OH)_2_, not in its ionic state^48^.

The impact of pH on the Pb(II) biosorption capability is displayed in Fig. 8b. It was noticed that the Pb(II) ions’ biosorption capacity elevated as the aqueous solution’s pH increased from pH 1.5 to 5, reaching its maximum value at a pH of 5. The competition between Pb(II) and H^+^ ions for sorption sites is the main reason adsorption decreases at lower pHs. On the other hand, as pH rises, more negatively charged surfaces become accessible, enabling greater metal uptake. Pb(II) is precipitated as its hydroxides at higher pH values (above 6), which slowed the rate of adsorption and, as a result, the proportion of metal ions removed.

#### Effect of biosorbent dose

Another crucial factor in Pb(II) sorption is the biosorbent dose. Using a Pb(II) concentration of 50 mg/L and different dosages ranging from 0.01 g to 0.09 g, the impact of the ANA/ANAS biosorbent dosage was investigated. Figure 8c illustrates that Pb(II) removal (%) using both ANA and ANAS samples decreased as the biosorbent dosage increased, indicating a decrease in adsorption capacity. As presented in Fig. 8c, the Pb(II) removal percentage rose from 40 and 50% at 0.01 g to reach 92.75% and 99.55% at 0.09 g for ANA and ANAS, respectively. Briefly, as ANA and ANAS dosage increases, the availability of larger active sorption sites increases, resulting in an elevation in Pb(II) ions sorption capability. Adsorption density decreases as the plant amount increases, due to the unsaturation of adsorption-active sites during adsorption^49^.

#### Effect of initial concentration of Pb(II)

**Fig. 9:**
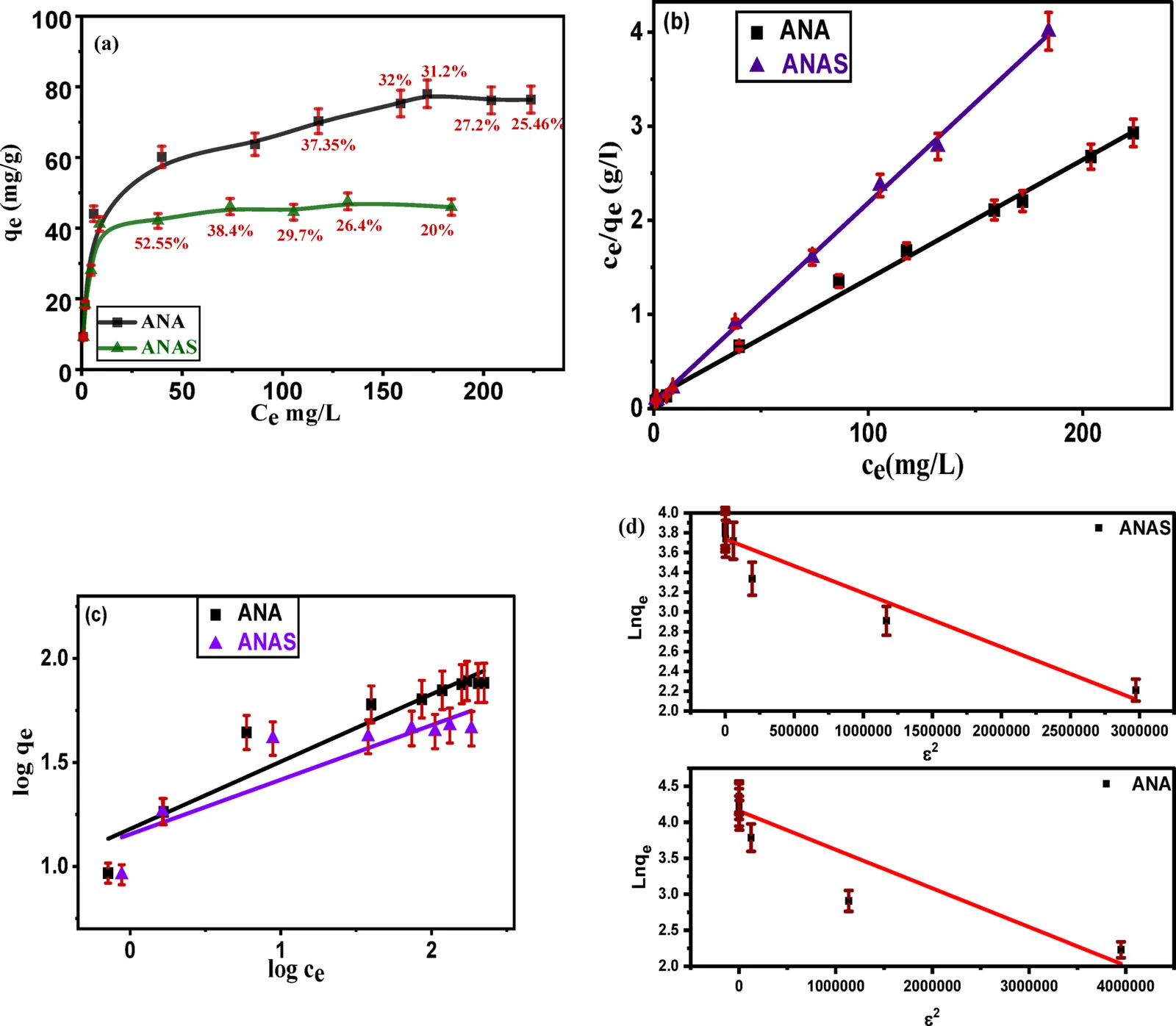
(**a**) Effect of initial Pb(II) concentration on adsorption capacity (conditions: 0.025 g of ANA/ANAS was taken at 25 mL of Pb(II) for 6 h at pH 4.5–5); (**b**) Langmuir adsorption isotherm model, (**c**) Freundlich adsorption isotherm model, and (**d**) D-R isotherm model, for Pb(II) biosorption using ANA and ANAS biosorbents.

Initial concentration is an essential parameter for estimating the effect of Sahara plants (ANA and ANAS) on Pb(II) removal. Figure 9a illustrates how the adsorption capacity increases with increasing Pb(II) concentration. This is illustrated by the fact that at lower Pb(II) concentrations, numerous adsorption sites are available. Furthermore, the available active sites on both ANA and ANAS biosorbents become saturated at higher Pb(II) concentrations, thereby reducing the plant’s removal (%) and biosorption capacity (mg/g) for Pb(II)^50^.

As the Pb(II) concentrations exceed the available ANA and ANAS biosorbents’ active sites, further Pb(II) biosorption is hindered, in addition to the stabilization of biosorption capacity, which results in a decreased Pb(II) remediation (%).

#### Biosorption isotherms

An equilibrium adsorption methodology shows the adsorbent’s capacity. The biosorbent surface features and affinity properties are expressed by a set of constant values that define an adsorption isotherm. These values can also be utilized to compare the biosorbent’s adsorptive capabilities for various contaminants^51^. Several adsorption isotherms have been used to evaluate the equilibrium kinetics data. Here, three isotherm models, viz. Langmuir, Freundlich, and Dubinin–Radushkevich (D–R) were applied to determine the nature of Pb(II) adsorption on the surface of Sahara samples in the solid–liquid equilibrium phase.

The Langmuir model presented in Eq. (12) was based on the assumption that the adsorbed layer was unimolecular; in addition to the adsorption process, it also accounted for chemical interactions.

12 $$\frac{{{\mathrm{c}}_{{\mathrm{e}}} }}{{{\mathrm{q}}_{{\mathrm{e}}} }} = \frac{1}{{{\mathrm{bq}}}} + \frac{{{\mathrm{c}}_{{\mathrm{e}}} }}{{\mathrm{q}}}$$

where C_e_ (mg/L) and b (L/mg) are the Pb(II) concentration at equilibrium and the Langmuir constant, respectively, additionally, q_e_ describes Pb(II) milligrams that were bio-sorbed per one gram of the ANA/ANAS biosorbent.

As shown in Fig. 9b, the C_e_/q_e_ is linearly plotted versus the C_e_, and q_e_ and b are determined. The favorable nature of Pb(II) biosorption may be identified utilizing Eq. (13) to estimate R_L_, the dimensionless separation factor of equilibrium.

13 $${\mathrm{R}}_{{\mathrm{L}}} = \frac{1}{{1 + {\mathrm{K}}_{{\mathrm{L}}} {\mathrm{C}}_{{\mathrm{O}}} }}$$

C_o_: is the highest initial concentration of the solute, K_L_: constant for the Langmuir model.

Irreversible isotherms can be classified as either favorable (0 < R_L_ < 1), linear (R_L_ = 1), unfavorable (R_L_ > 1), or when R_L_ = 0^23^. As shown in Table 3, the Pb(II) biosorption favourability can be evaluated as the R_L_ value is between (0.0118 and 0.028) within the range of (0 < R_L_ < 1), indicating that the Pb(II) biosorption process utilizing ANA and ANAS plants is favorable.

**Table 3:** Isotherm constants of Langmuir, Freundlich, and D-R for Pb(II) ions adsorption.

	Sahara samples	ANA	ANAS
Langmuir	q_m_ (mg/g) ± SD	79.05 ± 0.28	47.61 ± 0.19
	b (L\mg)	0.112	0.362
	R_L_	0.028	0.0118
	R^2^	0.995	0.998
	${x}^{2}$	132.53	58.922
	SSE	10,476.66	2805.296
	MSE	1047.66	311.699
	Hybrid	9550.26	3209.519
	$\Delta q\%$	757.59	260.974
Freundlich	K	15.13	14.12
	1/n	0.323	0.262
	R^2^	0.893	0.773
	${x}^{2}$	168.86	90.0288
	SSE	14,732.47	4984.662
	MSE	1473.24	553.851
	Hybrid	12,334.68	5034.589
	$\Delta q\%$	82,177.11	12,092.704
D-R	R^2^	0.934	0.839
	E (KJ/mole)	0.964	0.958

The Freundlich isotherm, whose linear form is given by Eq. (14), describes the heteroge­neous nature of the ANA biosorbent surface^52^.

14 $$\log {\mathrm{q}}_{{\mathrm{e}}} = \log {\mathrm{K}}_{{\mathrm{F}}} + \frac{1}{{\mathrm{n}}}\log {\mathrm{C}}_{{\mathrm{e}}}$$

K_F_ (L/mg): Freundlich constant (distribution coefficient, which indicates how much Pb(II) has been adsorbed onto the adsorbent for a single unit of equilibrium concentration).

n: Freundlich exponent (the value of 1/n is crucial for determining adsorption feasibility).

$\left( {{\mathrm{K}}_{{\mathrm{F}}} \,{\mathrm{and}}\,1{\mathrm{/n}}} \right)$ can be obtained from the intercept and slope of a linear regression between ${\mathrm{logq}}_{\mathrm{e}}$ Against $\mathrm{l}\mathrm{o}\mathrm{g}$ Cₑ as shown in Fig. 9c.

1/n values should be < 1 for the favorable adsorption procedure.

The Langmuir isotherm model, which proposes monolayer biosorption on a homogeneous biosorbent surface^27^, provides the best fit for Pb(II) biosorption onto the ANA and ANAS, as indicated by the highest R^2^ values (≥ 0.995) and the lowest error functions. The Langmuir isotherm was applied to estimate the monolayer adsorption capacities (q_m_) of Pb(II), yielding values of 79.05 mg/g for ANA and 47.61 mg/g for ANAS.

The Dubinin–Radushkevich (D–R) model examines adsorption from an energetic perspective and assumes that the process is associated with pore volume and surface porosity. Equation (15) defines the adsorption mean free energy (E_DR_) as determined by the D-R model. The value of E_DR_ indicates whether the adsorption process is chemical (8 < E_DR_ < 16 kJ mol^−1^) or physical (E_DR_ < 8 kJ mol^−1^). Where R is the gas constant (8.314 J/mol·K), and T is the temperature (in Kelvin). ε is the adsorption potential and is presented in Eq. (16).

15 $$E_{DR} = \frac{1}{{\sqrt {2K} }}$$

16 $${\upvarepsilon } = {\mathrm{RTln}}\left( {1 + \frac{1}{{{\mathrm{C}}_{{\mathrm{e}}} }}} \right)$$

With R^2^ values of 0.839 and 0.934, lower than those of Langmuir, the D-R isotherm model is not suitable for describing Pb(II) adsorption using ANA and ANAS, respectively.

#### Influence of contact time

**Fig. 10:**
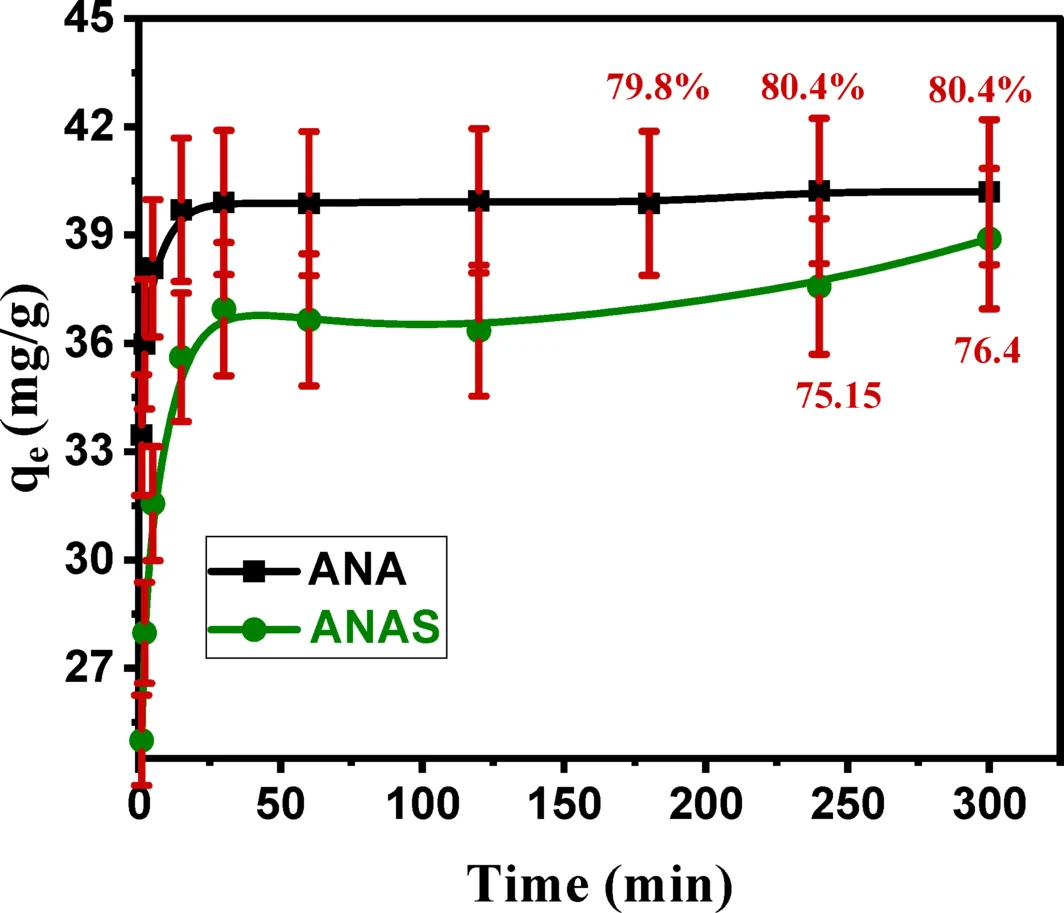
Effect of contact time on the biosorption of Pb(II) ions on the ANA an ANAS Sahara samples (conditions: 0.025 g of ANA/ANAS was taken at 25 mL, 50 mg/L of Pb(II) at pH 4.5–5).

The oscillation time parameter plays a crucial role in Pb(II) biosorption using ANA and ANAS biosorbents. This effect was investigated to estimate the duration required to achieve equilibrium. It was investigated over the range of (1–300 min) using (ANA and ANAS) samples and is presented in Fig. 10. In the first 30 min of Pb(II) shaking with ANA/ANAS biosorbent, the Pb(II) uptake was sharply increased, then became almost stable after 60 min, indicating reaching an equilibrium state. This might be linked to the abundance of active sites at high Pb(II) concentrations during the first phase. As time passes (around 60 min), fewer surface active sites on ANA and ANAS become available, which lowers the rate at which Pb(II) ions adsorb and, as a result, results in a lowering of metal uptake. As a result, the adsorbate (Pb(II) in solution and on the ANA/ANAS surface had greater concentration gradients. For this reason, the experimental methodology’s equilibrium time is set to 2 h to ensure equilibrium is reached.

#### Biosorption kinetic studies

Understanding biosorption kinetics is essential for evaluating the effectiveness and mechanisms of biosorption. Modeling adsorption kinetics helps identify suitable biosorbents and design optimal systems for removing pollutants, such as heavy metals, from various water samples under specific operational conditions. Moreover, pseudo-1^st^- and pseudo-2^nd^-order models were utilized to evaluate the rate kinetics of Pb(II) biosorption onto ANA & ANAS Sahara materials.

##### Pseudo-1^st^-order equation

Lagergren introduced a pseudo-first-order kinetic model, which has been widely used to describe the kinetics of various adsorption systems. Equation (17) shows the linearized form of this model^19^.

17 $$\log ({\mathrm{q}}_{{\mathrm{e}}} - {\mathrm{q}}_{{\mathrm{t}}} ) = \log {\mathrm{q}}_{{\mathrm{e}}} - \frac{{{\mathrm{k}}_{1} }}{2.303}{\mathrm{t}}$$

**Fig. 11:**
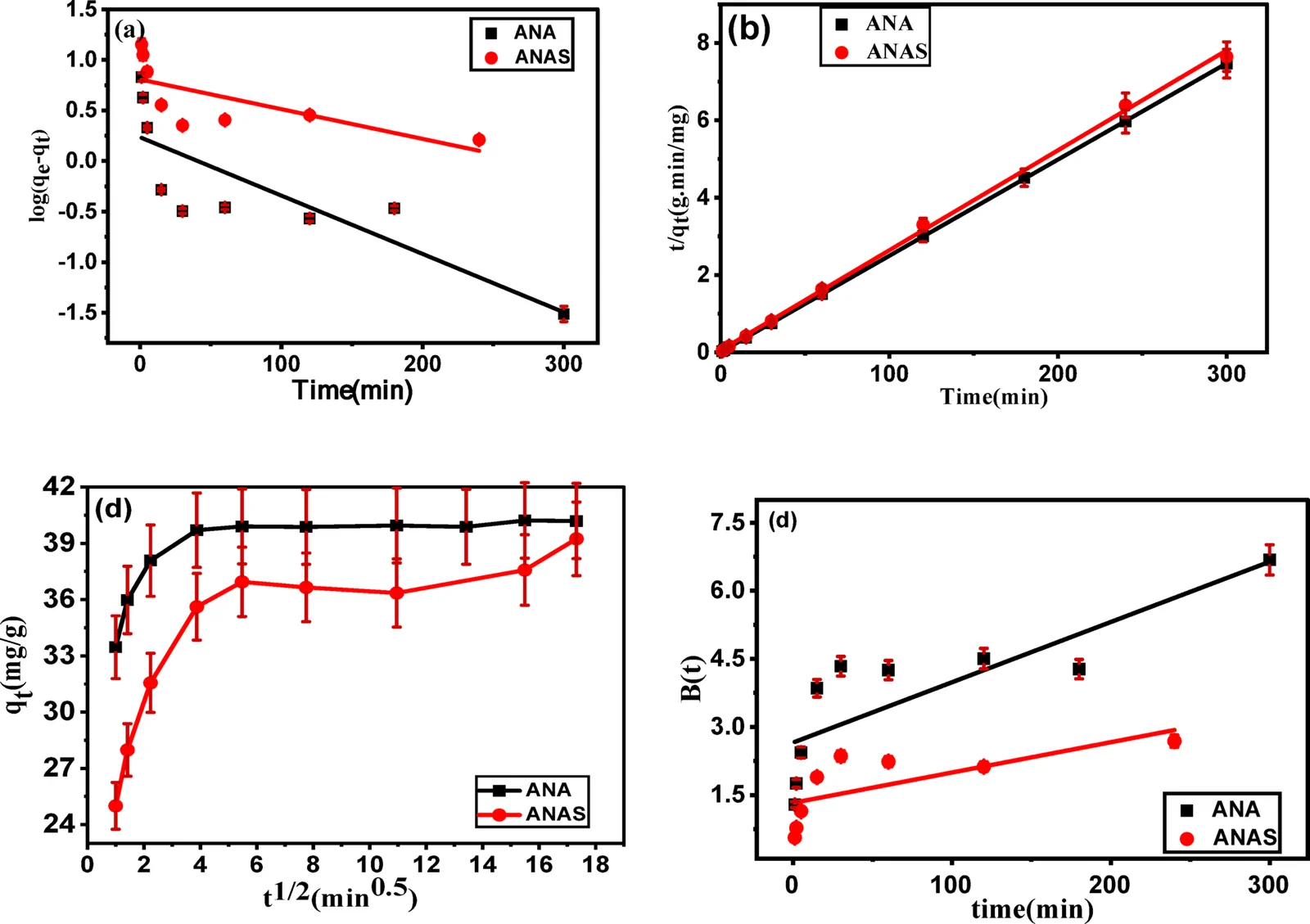
Kinetic models for the Pb(II) biosorption onto ANA and ANAS biosorbents: (**a**) Pseudo-1^st^-order, (**b**) Pseudo-2^nd^-order, (**c**) Intra-particle diffusion, and (**d**) Boyed model.

The parameters q_e_ (mg/g) and q_t_ (mg/g) indicate the quantities of Pb(II) biosorbed onto ANA and ANAS at equilibrium and at time t (min), respectively. The linear plot of log (q_e_–q_t_) versus t in Fig. 11(a) yields the values of K_1_ and q_e_ (mg/g) from the slope and intercept.

##### *Pseudo-2*^*nd*^*-order equation*

The biosorption kinetics can be described by the pseudo-2^nd^-order kinetic model, as shown in Eq. (18)^52^.

18 $$\frac{{\mathrm{t}}}{{{\mathrm{q}}_{{\mathrm{t}}} }} = \frac{1}{{{\mathrm{k}}_{2} {\mathrm{q}}_{{\mathrm{e}}}^{2} }} + \frac{1}{{{\mathrm{q}}_{{\mathrm{e}}} }}{\mathrm{t}}$$

K_2_ (g mg^-1^ min^-1^): the 2^nd^ rate constant.

The linear plot of t/q_t_ versus t, shown in Fig. 11(b), facilitates the determination of both K_2_ and q_e_. As indicated in Table 4 and Fig. 11(b), the correlation coefficient (R^2^) for the pseudo-second-order model ranges from 0.999 to 0.998 for ANA and ANAS, respectively. This value is significantly higher than that of the pseudo-first-order model, which ranges from 0.409 to 0.643 in the same order. Also, the agreement between the predicted q_e_ values and the experimental results indicates that the Pb(II) biosorption process likely follows a pseudo-2^nd^-order mechanism. Also, the error function values for the pseudo-2^nd^-order model, as presented in Table 4, are much lower than those for the pseudo-1^st^-order model. This means that Pb(II) binds to ANA and ANAS adsorbents in a manner consistent with the pseudo-2^nd^-order model, suggesting that a chemical reaction is involved^19^.

**Table 4:** Kinetic parameters for the Pb(II) biosorption onto ANA and ANAS biosorbents.

Plant samples	q_exp_ (mg/g) ± SD	ANA	ANAS
		40.22 ± 0.1	39.2 ± 0.12
Pseudo-1^st^-order kinetic model	q_1_ (mg/g)	1.718	6.409
	k_1_ (1/min)	0.0132	0.0677
	${R}_{1}^{2}$	0.643	0.409
	${x}^{2}$	7997.80	1106.123
	SSE	13,740.22	7089.145
	MSE	1374.022	787.682
	Hybride	4420.43	2894.592
	$\Delta q\%$	101.43	73.5668
Pseudo-2^nd^-order kinetic model	q_2_ (mg/g)	40.19	38.68
	k_2_ [g mg^-1^ min^-1^]	0.070	0.012
	${R}_{2}^{2}$	0.999	0.998
	${x}^{2}$	1.69	375.833
	SSE	68.049	9.7164
	MSE	6.80	41.759
	Hybride	24.72	197.6011
	$\Delta q\%$	0.6398	6.43
Intraparticle diffusion model	k_int_ [mg/(g. min^1/2^)]	0.039	1.317
	_C_	39.55	28.28
	${R}_{2}^{2}$	0.953	0.662
Boyd model	Intercept	2.65	1.327
	R^2^	0.415	0.125

##### Intra-particle diffusion (IPD)

The IPD presented in Eq. (19) is used to estimate diffusion mechanisms and to identify the rate-determining step in the adsorption process^27^.

19 $${\mathrm{q}}_{{\mathrm{t}}} = {\mathrm{k}}_{{{\mathrm{int}}}} t^{1/2} + {\mathrm{c}}$$

q_t_: the adsorbed Pb(II) amount onto ANA and ANAS (mg/g) at time (t), k_int:_ rate constant of the intra-particle diffusion model, C: determined from the intercept (it is constant).

If IPD is the rate-limiting step, plotting qt against t^1/2^ yields a straight line with slope k_int_. In some cases, both film diffusion and intraparticle diffusion affect adsorption kinetics.

It was shown that solute absorption frequently fluctuates nearly in proportion to t^1/2^ rather than to the contact time t in adsorption scenarios. Based on Eq. (17), q_t_ was plotted as a function of t^1/2^. If IPD is the rate-limiting step, the resulting plot should be linear, with a slope equal to k_int_. When IPD is the sole rate-limiting step, the qt versus t^1/2^ plot passes through the origin. Film diffusion and intraparticle diffusion may simultaneously affect adsorption kinetics. Therefore, the slope is nonzero, as shown in Fig. 11(c).

##### Boyd model

The kinetic expression developed by Boyd et al. was used to analyze the kinetic data further and determine whether Pb(II) biosorption proceeds via intraparticle or external diffusion, as shown in Eq. (20).

20 $${\mathrm{F}}\left( {\mathrm{t}} \right) = 1 - \frac{6}{{{\uppi }^{2} }}\mathop \sum \limits_{{{\mathrm{n}} = 1}}^{\infty } \frac{1}{{{\mathrm{n}}^{2} }}\exp \left( { - {\mathrm{n}}^{2} {\mathrm{Bt}}} \right)$$

In this context, F (as defined in Eq. (21)) represents the fraction of adsorbed solute at time t. The mathematical function of F is denoted as B(t), and n is an integer that characterizes the terms in the infinite series solution.

21 $${\mathrm{F}} = \frac{{{\mathrm{q}}_{{\mathrm{t}}} }}{{{\mathrm{q}}_{{\mathrm{q}}} }}$$

Both q_e_ and q_t_, measured in mg/g, represent the quantities of Pb(II) biosorbed onto ANA or ANAS biosorbents at the optimum time and at time t (min), respectively.

Reichenberg reported in Eqs. (22) and (23)^53^:

22 $${\mathrm{For}}\,{\mathrm{F}}\,{\mathrm{values}}\, > \, 0.{85};\quad {\mathrm{B}}\left( {\mathrm{t}} \right) = - 0.{4977} - {\mathrm{ln}}\,\left( {{1} - {\mathrm{F}}} \right)$$

23 $${\mathrm{For}}\,{\mathrm{F}}\,{\text{values < }} 0.85; \quad B \left( t \right) = \sqrt \pi \sqrt {\pi - \left( {\frac{{\pi^{2} F\left( t \right)}}{3}} \right)}$$

It is evident from Fig. 11(d) that the linear plots of this model do not pass through the origin, indicating that both film diffusion and intraparticle diffusion contribute to the rate-determining step. However, Fig. 11(d) (Boyd model) shows that the film-diffusion phase is the rate-determining step in Pb(II) biosorption, as the linear plots do not intersect the origin.

#### Statistical error validity

From the error functions, we can estimate which model provides a better fit to Pb(II) biosorption onto these novel Sahara plants. The best-fitting model to the experimental data is the one with a higher R^2^ and lower function errors. Table 3 indicates that the Langmuir isotherm provides the most accurate biosorption model, as evidenced by its higher R^2^ value and lower error metrics relative to the Freundlich isotherm. Also, the validation of the kinetic model is based on the data from Table 4. The 2^nd^-order kinetic model is more suitable for the current adsorption process, as it yields a higher R^2^ value and lower error function values than the Pseudo-1^st^-order model^54^.

#### Effect of temperature and thermodynamic studies

**Fig. 12:**
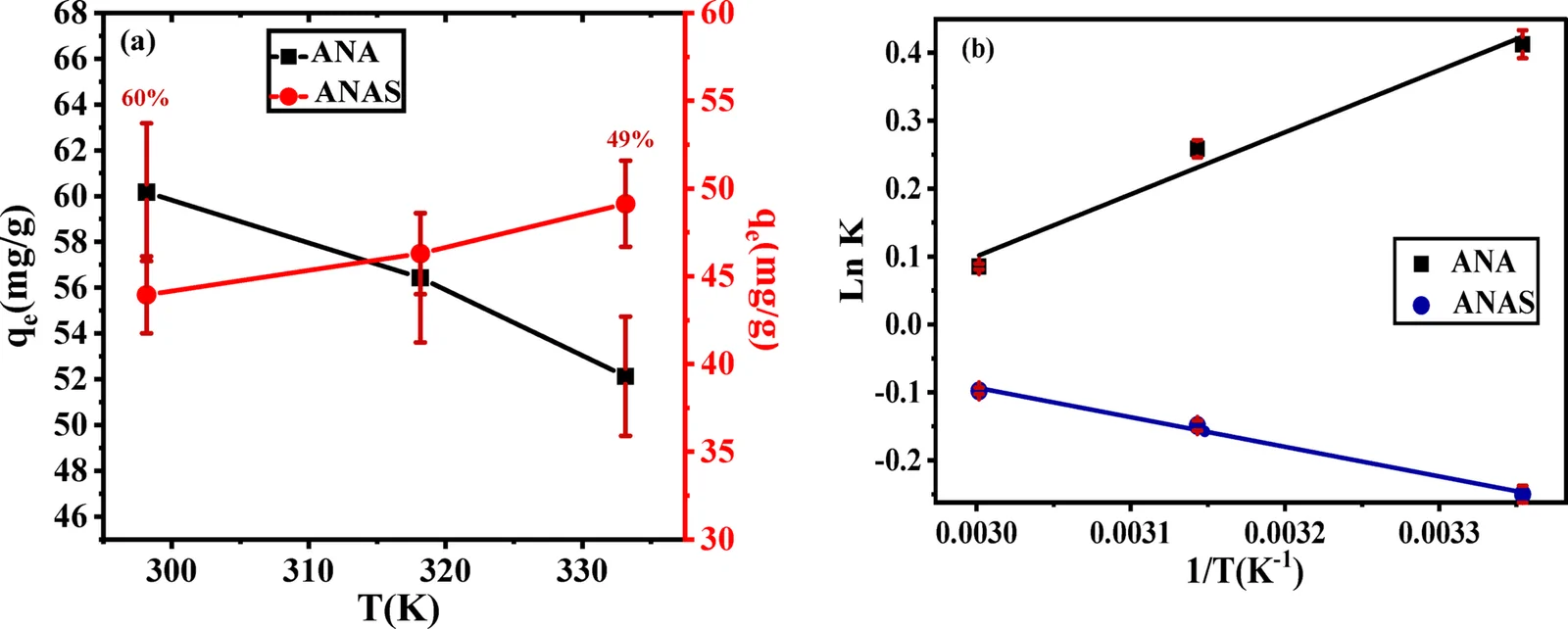
(**a**) Effect of temperature on the Pb(II) biosorption capacity on ANA and ANAS biosorbents (conditions: 0.025 g of ANA/ANAS; 25 mL 100 mg/L of Pb(II) for 2 h; pH 4.5–5); (**b**) Plot of ln K against (1/T) for the adsorption of Pb(II) on ANA and ANAS biosorbents.

Figure 12a shows that the adsorption capacity of ANAS increases with increasing temperature, indicating that the biosorption process is endothermic. In contrast, Pb(II) biosorption onto ANAS is more efficient at lower temperatures. This can be explained by the adsorption thermodynamics, which will be discussed later. In the case of ANA, adsorption capacity decreases with increasing temperature, indicating that Pb(II) biosorption onto ANA does not require high energy and can be explained by adsorption thermodynamics; it may also be due to intermolecular pore diffusion^55^.

By evaluating the thermodynamic parameters, the Pb(II) biosorption nature on the ANA or ANAS biosorbents was predicted, including biosorption standard free energy (ΔG^o^_ads_), heat enthalpy (ΔH^o^_ads_), and entropy (ΔS^o^_ads_).

The enthalpy (∆H^o^) and entropy change (∆S^o^) values were calculated using the slope and intercept of Eq. (24), respectively^23^:

24 $$\ln {\mathrm{K}}_{{\mathrm{c}}} = \frac{{ - \Delta {\mathrm{H}}^{{\mathrm{o}}} }}{{{\mathrm{RT}}}} + \frac{{\Delta {\mathrm{S}}^{{\mathrm{O}}} }}{{\mathrm{R}}}$$

R: universal gas constant, valued at 8.314 × 10^−3^ kJ per mole per kelvin. T: temperature, measured in kelvin (K).

The Pb(II) biosorption using ANA and ANAS biosorbents was spontaneous and feasible, as indicated by the thermodynamic variables investigated. Table 5 and Fig. 12b show that the Pb(II) adsorption is thermodynamically possible and spontaneous due to the negative ΔG^o^ value for the ANA sample. Elevated temperatures enhance the adsorption of the ANAS sample, as evidenced by the negative, relatively lower ΔG^o^ value. A positive ∆H^o^ value indicates an endothermic adsorption process, as evidenced by increased Pb(II) uptake at higher temperatures.

**Table 5:** Thermodynamic parameters for the adsorption of Pb(II) on ANA and ANAS samples.

Sample	K_c_			ΔG^0^ (kJ/mole)			ΔH^0^ (kJ/mole)	ΔS^0^ (J/mole K)
	298.15 K	318.15 K	333.15 K	298.15 K	318.15 K	333.15 K		
ANA	1.5106	1.2951	1.0889	− 1.022	− 0.683	− 0.235	− 7.59	− 21.95
ANAS	0.7789	0.8618	0.9069	0.61939	0.39341	0.27067	3.62	10.09

The exothermic nature of Pb(II) biosorption on the ANA biosorbent is demonstrated by the negative ∆H^o^ value. The negative ∆S^o^ values indicate a decrease in randomness at the solid (ANA)/Pb(II) solution interface during Pb(II) adsorption.

For ANAS, a positive ∆H^o^ value indicates that the biosorption process is endothermic, leading to increased Pb(II) uptake with increasing temperature^56^. A positive value of ∆S^o^ was observed in the ANAS sample, indicating increased randomness at the adsorbate-adsorbent interface for Pb(II) adsorption, which may be attributed to the change in the adsorbent surface structure after the treatment with NaOH.

#### Effect of ionic strength

Researchers have examined the impact of ionic strength on metal ion adsorption by analyzing changes in solute activity and variations in the thickness of the diffuse electrical double layer. The electrical double layer configuration of the biosorbent surface and the aqueous metal chemistry are both altered by ionic strength, which also affects the adsorption of metal ions. The effect was examined using ANA and ANAS samples at a Pb(II) concentration of 20 mg/L, with NaCl concentrations ranging from 1 × 10⁻^3^ M to 0.15 M at neutral pH to avoid interference from the buffer solution. Figure S2 shows that Pb(II) removal efficiency declines as NaCl concentration increases. This reduction is attributed to competition between Pb(II) ions and Na^+^ cations for the active sites of ANA and ANAS biosorbents^57,58^.

#### Effect of foreign ions

Naturally occurring water anions and cations present in various water sources significantly affect heavy metal removal. The impact of these ions on Pb(II) biosorption was studied using 100 μg/mL of each ion, such as fluoride, chloride, and acetate, Ca^2+^, KH_4_^+^, and Mg^2+^, which may be present in various water resources. As shown in Table 6, under optimal adsorption conditions, Pb(II) remediation is only slightly affected by the investigated foreign ions. From the results observed in Table 6, both ANA and ANAS biosorbents demonstrate high efficiency in removing Pb(II) ions, even in the presence of diverse water anions and cations.

**Table 6:** The different interfering cations and anions’ impact on the Pb(II) removal (R, %) using ANA and ANAS biosorbents.

Plant samples	ANA	ANAS
Foreign ions	R (%) ± SD	
Acetate^-^	93.26 ± 0.17	96.05 ± 0.14
Cl^-^	93.47 ± 0.05	96.85 ± 0.23
F^-^	82.45 ± 0.39	96.15 ± 0.16
Po_4_^3-^	94.5 ± 0.24	95.9 ± 0.12
NH_4_^+^	92.03 ± 0.11	94.65 ± 0.31
Na^+^	94.64 ± 0.33	95.2 ± 0.17
Ba^2+^	91.89 ± 0.15	94 ± 0.09
Mg^2+^	58.95 ± 0.13	66.8 ± 0.22

#### Desorption studies

ANAS biosorbent can be economically repurposed using various techniques to regenerate the sample. Regeneration is based on the stability of these biosorbents, which can withstand temperature changes and resist both basic and acidic conditions. Reusing or regenerating such biosorbents in situ has become more attractive due to rising environmental consciousness and the costs of disposing of contaminated bioresidues. There are several benefits to regeneration. There are several advantages of regeneration. It preserves landfill space and lessens the environmental impact of disposing of used biosorbents.

Desorption studies were conducted by varying the solution pH from 1.5 to 10 using HCl and NaOH to determine whether the generated activated plants could be reused. Their capacity to desorb from the loaded pollutants is a measure of their commercial efficiency. Figure S3 depicts the desorption effect. As the solution’s pH increased, the desorption efficiency reduced.

The desorption efficiency decreased with increasing pH of the Pb(II) solution. For the ANAS sample, desorption efficiency reached 73.53% at pH 1.5 and decreased sharply to 2.55% at pH 10. These findings demonstrate that Pb(II) desorption from the ANAS sample after the biosorption step can readily occur. Thus, the ANAS biosorbent can be effectively reused for Pb(II)- contaminated water purification^19^.

### Analytical applications

Environmental samples, including tap, deionized, and groundwater, as well as digested food samples, were investigated to assess the ability of these plant biosorbents to remove Pb(II) ions. Each sample (25 mL) was spiked with Pb(II) at concentrations ranging from 5 to 15 mg/L under optimal conditions (pH 5, 25 °C). For food samples, 25 mL of the investigated sample was spiked with 20 mg/L of Pb(II). The solutions were then mixed with 0.025 g of the biosorbent under study. The filtrate was analyzed by atomic absorption spectroscopy. The data in Table 7 shows that the recovery for water samples spiked at the concentration was 97.8–100%. As the spiked concentration increased, recovery decreased. ANAS achieved a recovery of 31.32:44.07%. Generally, the performance was satisfactory, indicating that these sorbents could be employed with high accuracy and precision to detect and eliminate Pb(II) ions in real samples.

**Table 7:** Pb(II) metal ion recovery from different environmental samples using prepared adsorbents (*n* = 3).

Environmental sample	Bio-sorbents		ANA		ANAS	
	Pb(II) added in (mg/L)	Measured after spiking	Recovery (R, %) ± SD	RSD(%)	Recovery (R, %) ± SD	RSD(%)
Deionized water	0	0	–	–	–	–
	5	5.02	98.5 ± 0.14	0.14	97.8 ± 0.09	0.09
	10	9.98	92.2 ± 0.6	0.65	91.21 ± 0.16	0.18
	15	15	91.5 ± 0.27	0.3	90.35 ± 0.56	0.62
Tap water	0	0.1	–	–	–	–
	5	5.09	88.16 ± 0. 27	0.31	96.9 ± 0.23	0.24
	10	10.1	51.3 ± 0.06	0.11	58.3 ± 0.06	0.11
	15	15.07	20 ± 0.19	0.94	39.93 ± 0.06	0.16
Ground water	0	0.14	–	–	–	–
	5	5.11	96.44 ± 0. 16	0.17	100 ± 0.05	0.05
	10	10.16	91.94 ± 0.07	0.08	85.4 ± 0.18	0.21
	15	15.08	28.86 ± 0.36	1.25	74.82 ± 0.07	0.09
Flour	20	20.04	–	–	31.32 ± 0.23	0.75
Rice	20	19.97	–	–	39 ± 0.25	0.64
Herbal extract	20.1	20.01	–	–	44.07 ± 0.38	0.87

### Performance of the ANA and ANAS Sahara plants-derived biosorbents

Previous investigations have studied many biosorbents for their capacity and efficiency in eliminating Pb(II). Here, we employed *A. articulata* in both raw and mercerized forms as efficient biosorbents for Pb(II) removal. Comparing the investigated materials, ANA and ANAS, with other materials from published studies shows several clear benefits: (i) Environmental sustainability: ANA, as an agro-waste material, represents an environmentally sustainable option for the biosorption process. Utilizing agricultural waste helps mitigate disposal challenges and supports sustainable methods for treating heavy metals. (ii) Cost-efficiency: Due to its lower cost compared to conventional adsorbents, ANA represents a practical option for industrial applications, particularly in developing countries with limited financial resources. iii. High Adsorption Capacity: According to preliminary findings, ANA and ANAS have Pb(II) adsorption capacities that are on par with, or even exceed, those of other adsorbents, such as coconut coir and pokeweed (Table 8). This phenomenon results from the porous structure of the ANA biosorbent and the presence of multiple functional groups, which enhance Pb(II) binding. iv. Availability: due to its widespread availability, ANA can be more relevant across a variety of geographic settings and can serve as a viable option for removing heavy metals from wastewater treatment.

**Table 8:** Pb(II) maximum sorption capacity of the proposed derived bio-sorbent compared with previously published biosorbents.

Biosorbent	Initial concentration mg/L	q_e,_ mg/g	Maximum R (%)	Biosorbent type	Ref
H_2_O_2_-modified watermelon seeds biochar	100	60.78	–	Plant	^59^
KOH-modified banana peel hydrothermal carbon (K-HTC)	50	42.92	86.84	Plant	^60^
Phosphoric-acid modified banana peel hydrothermal carbon	100	40.64	82.74	Plant	^61^
Pokeweed (untreated)	5–300	10.83	81.97	Plant	^62^
Pokeweed (treated with nitric acid)		12.66	93.29	Plant	^62^
Durian tree sawdust	200	20.37	40.74	Plant	^63^
Coconut coir		37.04	74.08	Plant	^63^
Oil palm empty fruit bunch		37.59	75.18	Plant	^63^
Olive tree pruning (untreated)	150	27.05	–	Plant	^43^
ANA	50	76.38	100	Plant	Present study
ANAS	50	46.74	93.48	Plant	Present study

As shown in Table 8, a comparative investigation of the maximum Pb(II) removal capacity (q_e_) of various affordable plant-derived biosorbents was conducted. Compared to synthetic resins, these biosorbents are more affordable and more accessible. The estimated q_e_ values indicate that ANA and ANAS are effective in biosorbing Pb(II). This result emphasizes the efficiency and environmental friendliness of the biosorbent ANA for treating Pb(II) contamination in wastewater.

### Plausible mechanism of biosorption of Pb(II) onto ANA and ANAS biosorbents

**Fig. 13:**
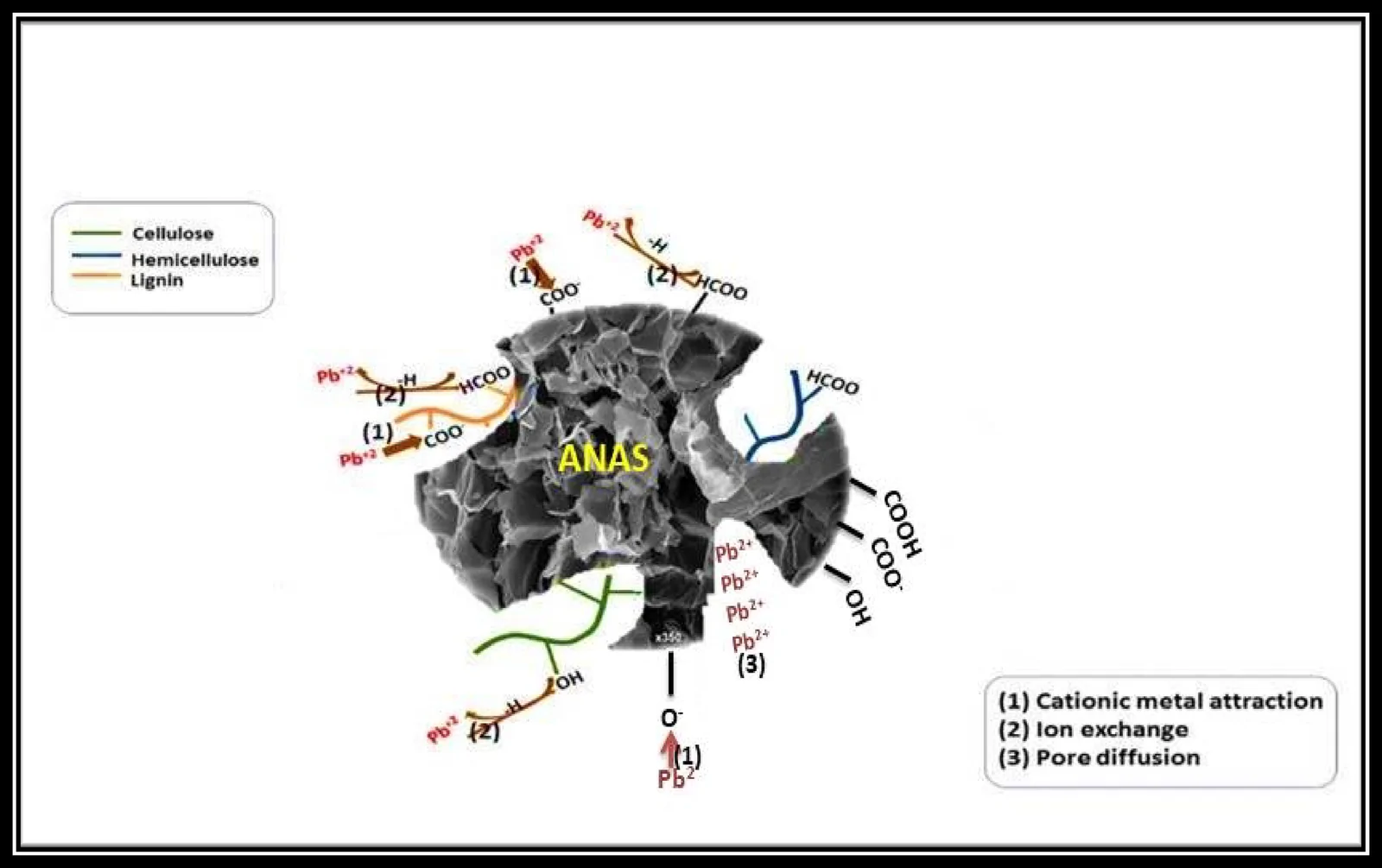
Plausible mechanism of adsorption of Pb(II) onto *A. articulata* Sahara plant derived biosorbent(ANAS).

The process by which ANA and ANAS biosorb Pb(II) is thoroughly unraveled, revealing the intricate steps involved using a cooperative approach that combines theoretical computations with experimental analysis. Figure 13 illustrates this complex system. The Pb(II) biosorption onto ANA and ANAS biosorbents is influenced by various factors, including isotherm and kinetic investigations, pH effects, and FTIR. The main Pb(II) biosorption mechanisms for raw and mercerized ANA and ANAS, respectively, include^64–66^: (i) Pore diffusion: The radius of Pb(II) is (46 Å) smaller than that of the pore diameter of ANA and ANAS (73.4 and 130.5 Å), which allows the easy pore diffusion of Pb(II) into ANA and ANAS; (ii) Ion exchange: The surfaces of both ANA and ANAS contain various acidic groups, such as carboxyl and phenolic hydroxyl, which are capable of forming complexes with Pb(II); (iii) Electrostatic attractions: Where positive charges of Pb(II) interact with the ANA and ANAS negative charges. Electrostatic adsorption occurs when ionic bonds form, which happens when the pH exceeds the pH_PZC_ of ANA and ANAS. A comprehensive interpretation of biosorption provides valuable insights that deepen fundamental understanding and facilitate the optimization and customization of biosorption strategies for remediating Pb(II)-contaminated waters. These discoveries, depicted in Fig. 13, enable the development of more effective and durable remediation techniques and provide insight into the complex mechanism underlying Pb(II) biosorption using the biosorbents ANA and ANAS.

Surface chemistry significantly influences Pb(II) adsorption. The Langmuir isotherm analysis indicates that adsorption by both ANA and ANAS predominantly occurs via a monolayer process, which does not exclusively imply a physical adsorption mechanism. Chemical interactions, including ion exchange and complexation, are likely to contribute to the overall mechanism of Pb(II) adsorption. NaOH treatment did not result in a consistent increase in Pb(II) adsorption capacity relative to the untreated biosorbent (ANA). Rather, NaOH treatment primarily modified the surface chemistry and redistributed functional groups, as demonstrated by FTIR and Boehm titration analyses.

### Environmental impact

Choosing natural materials like ANA as biosorbents for removing water contaminants often results in a gentler, more positive environmental impact than relying on conventional chemicals^67^. These natural materials are environmentally benign and renewable. Furthermore, prior to application, the properties and environmental performance of the materials are assessed using computational techniques, including molecular modeling. The overwhelming majority of utilized metal adsorbents are considered toxic substances. Natural biosorbents must replace these materials due to growing environmental protection regulations and global demands for environmental consciousness. This investigation focuses on ANA and ANAS as new eco-friendly environmental biosorbents for Pb(II) elimination.

### Compliance with regulations

The application *A. articulata*, in its raw (ANA) and mercerized (ANAS) forms, serves as a biosorbent for removing heavy metals, including Pb(II), meeting both local and global environmental standards. Several important regulatory frameworks and recommendations are ensured to be followed by our research:

1. Local environmental legislation: Local environmental protection organizations have established national requirements, which we have followed. To ensure that the techniques used do not release additional pollutants into the environment, these regulations usually include recommendations on the use of natural materials in wastewater treatment processes. *A. articulata* in both forms, raw and mercerized ones, does not emit any dangerous compounds during or after the adsorption process, according to thorough evaluations and toxicity studies.
2. Global guidelines and standards: Our approach aligns with international standards, including the influential Water Framework Directive (WFD) of the European Union and the comprehensive guidelines issued by the Environmental Protection Agency (EPA). The use of ANA and ANAS as biosorbents is supported by ANA’s natural origin, biodegradability, and non-toxicity, which meet the strict requirements set by these organizations.

In conclusion, ANA and ANAS, when applied as biosorbents, comply with all applicable local and international environmental regulations. This observance assures that our method is not only successful and effective, but also ecologically friendly and sustainable.

### Practical applications and future research prospects

The ANA and ANAS biosorbents demonstrate low cost, straightforward preparation, competitive adsorption capacities, and a wide range of raw material sources, suggesting their suitability for Pb(II) removal from aqueous solutions. Future research should investigate additional Sahara plant sources to expand the availability of sustainable, low-cost biosorbent materials. Furthermore, elucidating the molecular-scale interactions between heavy metal ions and ANA/ANAS will provide valuable insights for the design of more effective biosorbents.

### Alignment of ANA and ANAS biosorbents with green chemistry principles

ANA and ANAS adhere to several key principles of green chemistry. First, both utilize renewable feedstocks by employing raw biomass from a locally abundant desert plant, thereby reducing reliance on consumable and non-renewable resources. The principle of safer chemical synthesis is addressed by mercerizing ANAS under mild conditions with NaOH, thereby avoiding the use of hazardous reagents. Energy efficiency is achieved because the ANAS modification occurs at relatively low temperatures, consuming less energy than conventional adsorbent preparation methods. The design meets the degradation and reusability principles, as both ANA and ANAS serve as biodegradable biosorbents that can be reused multiple times, minimizing environmental impact. The integration of various functionalities is demonstrated by their ability to remove Pb(II) and exhibit antioxidant and antimicrobial properties, expanding their applicability in environmental and biomedical contexts. Finally, practical application in real wastewater is validated, as ANA and ANAS have been tested on various real wastewater sources, confirming their versatility and suitability for real-world implementation.

## Biological activity studies of *Anabasis articulata* Sahara plants

### Antioxidant activities

In human bodies, Free radicals (also known as reactive oxygen species (ROS) and nitrogen active species (RNS)) are chemical species that are formed and thought to be able to take electrons from other radicals. These free radicals can damage cells’ DNA, lipids, and proteins, leading to harmful health effects, including diabetes mellitus and cancer. Antioxidants are defined as substances that prevent or scavenge free radicals, thereby reducing tissue damage^68^. In recent years, plant extracts have been used as natural antioxidant sources that can scavenge free radicals and reduce damage to biomaterials such as proteins and DNA^69^.

The *A. articulata* plant is regarded as a medicinal plant because of the nature of its compositions, so it is tested against antioxidant activities. The DPPH assay, which employs 2,2-diphenyl-1-picrylhydrazyl (Fig. S4^69^), a compound known for its stable free radical properties, was used in this method.

**Fig. 14:**
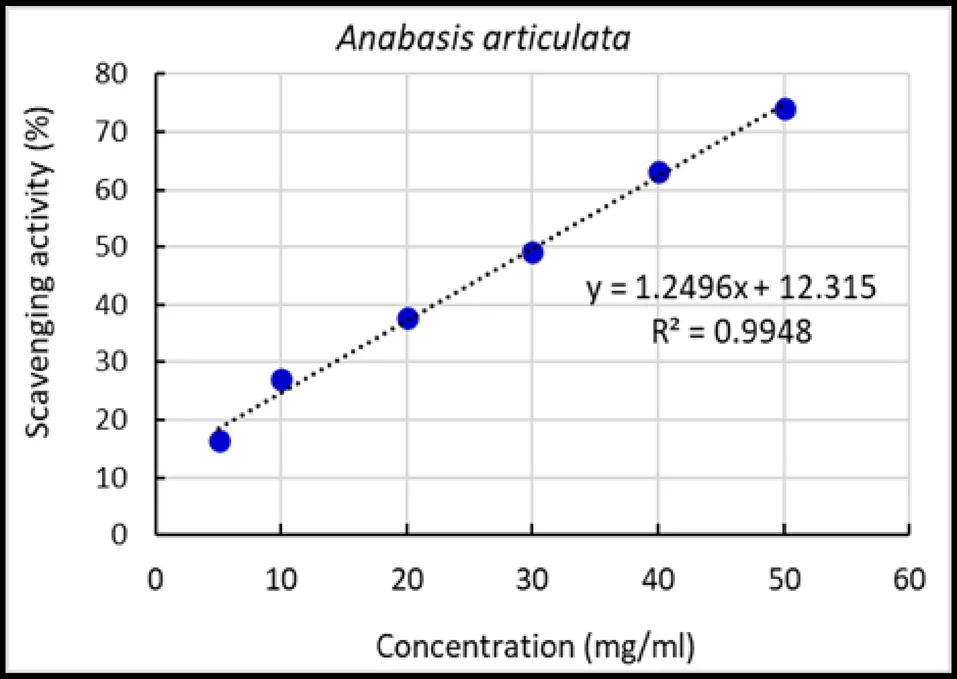
The standard curves of % of scavenging activities versus sample concentrations.

According to the data in Table 9 and Fig. 14, the *A. articulata* metabolic extract exhibited antioxidant activity compared to the standard reference Ascorbic acid. At 5 mg/mL, the extracts of *A. articulata* showed 16.32% scavenging activity. However, as the concentration of the plant extracts increases, the scavenging activity increases, reaching 74.05% at 50 mg/mL for *A. articulata* extracts. With IC_50_ values ranging from 10 to 50 mg/mL, the plant extracts are considered to pack a powerful antioxidant punch^70^.

**Table 9:** The DPPH Scavenging activity (%), LSD_0.05_, and IC_50_ (mg/mL) values of ANA ethanolic extract and ascorbic acid as a standard.

Conc. (mg/mL)	Scavenging activity* (%)	Conc. (mg/mL)	Scavenging activity* (%)
	*A. articulata*		Ascorbic acid
5	16.32 ±	1	2.73 ± 0.09
10	27.14 ±	2.5	10.99 ± 0.35
20	37.68 ±	5	38.20 ± 0.75
30	49.17 ±	10	45.19 ± 0.82
40	63.21 ±	15	56.23 ± 1.23
50	74.05 ±	20	64.97 ± 1.52
IC_50_ mg/mL	30.16	IC_50_ mg/mL	13.32
LSD_0.05_	0.0004***	LSD_0.05_	1.73***

*Values are means of triplicate ± standard error, IC_50_: the sample concentration needed to result in a 50% decrease in DPPH absorbance.

*A. articulata* methanolic extract stood out for its remarkable antioxidant activity, boasting IC50 values of 30.16 mg/mL and 45.36 mg/mL. These findings highlight the plant’s impressive antioxidant power.

### Antimicrobial bioassay

Many plant species have been recognized as potential antimicrobial agents for treating infectious diseases caused by various microorganisms. Antimicrobial agents can be exemplified by plants that are abundant in phenolic chemicals, alkaloids, and flavonoids.

#### Antibacterial activity assay

**Fig. 15:**
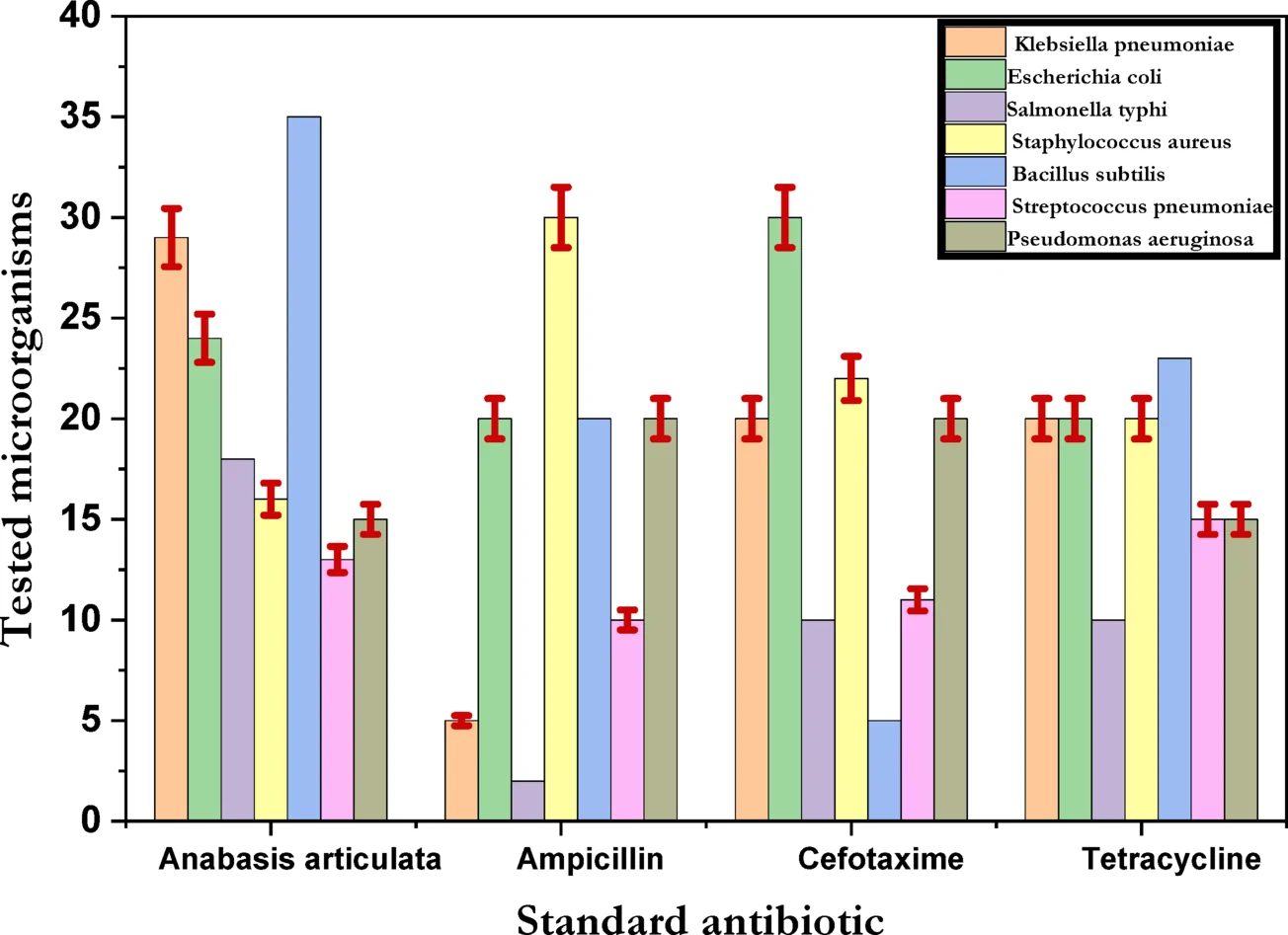
Distribution of inhibition zones using raw *A. articulata*.

The inhibition zone diameter (mm) produced by ANA alcoholic extracts against the tested bacteria is used to assess the investigated plant’s (ANA) antimicrobial activity. As shown in Table 10 and Fig. 15, the inhibition zone varies depending on the type of bacteria examined and the plant extracts. *Tetracycline*, *Cefotaxime*, and *Ampicillin* are the three common antibiotics to which these outcomes are compared.

**Table 10:** Antibacterial activity of the alcoholic extract from the aerial parts of *A. articulata* (ANA) and certain selected antibiotics as references at a concentration of 10 mg/mL.

Tested microorganisms	MeOH extracts (10 mg/mL)	Standard antibiotic (10 mg/mL)		
	*A. articulata*	*Ampicillin*	*Cefotaxime*	*Tetracycline*
Gram-negative bacteria				
*Klebsiella pneumoniae*	29	5	20	20
*Escherichia coli*	24	20	30	20
*Salmonella typhi*	18	2	10	10
*Pseudomonas aeruginosa*	15	20	20	15
Gram-positive bacteria				
*Staphylococcus* *aureus*	16	30	22	20
*Bacillus* *subtilis*	35	20	5	23
*Streptococcus* *pneumoniae*	13	10	11	15

*In case of Gram-negative bacteria*: In the case of *A. articulata* extract, it was the sturdiest inhibitor against *Klebsiella pneumonia* (29 mm), followed by *Escherichia coli* (24 mm), *Salmonella typhi* (18 mm), and finally *Pseudomonas aeruginosa* (15 mm).

*In case of Gram-positive bacteria*: In the case of *A. articulata* extract, they were the sturdiest inhibitors vs. *Bacillus subtilis* (35 mm), followed by *S. aureus* (16 mm), and finally *Str. pneumonia* (13 mm).

When we compare the data obtained from plant extract and antibiotics, we can observe that the plant extract has a good antimicrobial effect, and in some cases, it exhibits antimicrobial effects more than antibiotics, as in the following cases:

- Plant extracts exhibit antimicrobial activity against *Klebsiella pneumoniae* and *Salmonella typhi* that exceeds that of standard antibiotics. Also, *A. articulata* has a potent effect against *Escherichia coli* (24 mm), more than *Ampicillin* and *Tetracycline* (20 mm).
- Plant extract exhibits stronger antimicrobial activity against Bacillus subtilis than the standard antibiotics. *A. articulata* extract against *Streptococcus pneumoniae* (13 mm) has more antimicrobial effects than *Ampicillin* and *Cefotaxime* (10, 11 mm, respectively).

## Conclusion

This investigation explores the potential of the *A. articulata* Sahara plant in two forms, ANA and ANAS, as highly effective, eco-friendly, and low-cost biosorbents for Pb(II) remediation from diverse water sources. Moreover, efficient Pb(II) removal is typically achieved at pH 4.5 and after 60 min of oscillation, with maximum biosorption capacities of 76.38 and 46.74 mg/g, respectively. The experimental data closely fit the Langmuir isotherm and the Pseudo-2^nd^-Order kinetic model, indicating that Pb(II) biosorption by ANA and ANAS occurs via monolayer formation and chemisorption. Thermodynamic investigations conducted over the temperature range of 25 to 60 °C confirmed the endothermic nature of biosorption by ANA and ANAS biosorbents. The results of the regeneration experiments indicate that ANAS biosorbents exhibit substantial reusability. The valuable antimicrobial and antioxidant properties of *A. articulata* were demonstrated, as it is rich in bioactive 2-ry metabolites, including phenolics and flavonoids. Gallic acid, chlorogenic acid, and rutin are effective in neutralizing free radicals, as demonstrated by the DPPH assay. Pb(II) biosorption occurs through cationic metal attraction, ion exchange, and pore diffusion. Finally, this study highlights an effective biosorbent, rarely reported in the literature for Pb(II) removal via biosorption, due to its low cost, natural abundance, and efficient performance. Future studies could investigate their effectiveness for other hazardous metals, their performance in actual wastewater matrices, and the feasibility of scaling up for continuous treatment systems.

## Supplementary Information

Below is the link to the electronic supplementary material.

Supplementary Material 1

## Data Availability

All data generated or analysed during this study are included in this published article.
