# Supplementary material for: Valorizing the biosorptive potential and bioactivity of Anabasis articulata Sahara plants
Source: Sci Rep. 2026 Aug 20;16:26200. doi: 10.1038/s41598-026-66204-x (PMC13494034; doi:10.1038/s41598-026-66204-x)
Supplement: Supplementary file 1 [file 41598_2026_66204_MOESM1_ESM.docx]

**Supplementary data**

**Valorizing the biosorptive potential and bioactivity of *Anabasis articulata* Sahara Plants**

Magda A. Akl^1*^, ,Asmaa A Serage^1^, Aya G Mostafa^1^, Ahmed I Abd-Elhamid^3^, and Yasser Al-Amier^2^

^1^Department of Chemistry, Faculty of Science, Mansoura University, Mansoura 35516, Egypt

^2^Botany Department, Faculty of Science, Mansoura University, Mansoura 35516, Egypt

^3^Composites and Nanostructured Materials Research Department, Advanced Technology and New Materials Research Institute (ATNMRI), City of Scientific Research and Technological Applications (SRTA-City), Alexandria, New Borg Al-Arab, 21934, Egypt.

**Corresponding author:**

Prof Magda A Akl.

e-mail magdaakl@yahoo.com

**Table S1.** Optimization of Graphite Furnace Atomic Absorption Spectrometry.

| **Element** | **Pb(II)** |
| --- | --- |
| Lamp current (mA) | 10.0 |
| Wavelength (nm) | 283.3 |
| Slit width (nm) | 0.5 |
| Measurement mode | Peak Height |
| Sample volume | 20 µl |
| Ar rate flow (L/min) | 0.3 |

|  |  |
| --- | --- |

**Fig. S1:** *A. articulata* plant.

**Fig. S2:** Ionic strength impact on the Pb(II) remediation utilizing (ANA&ANAS) samples (conditions: 0.025 g of ANA/ANAS was taken at 25 mL 20 mg/L of Pb(II) for 24 h).

**Fig. S3:** Desorption of Pb(II) from ANAS by altering the pH values of the solution using HCl and NaOH.

**Fig. S4:** Mechanism of DPPH free radical scavenging activity **[84].**
